# Supplementary material for: Experimental manifestation of redox-conductivity in metal-organic frameworks and its implication for semiconductor/insulator switching
Source: Nat Commun. 2023 Jul 20;14:4388. doi: 10.1038/s41467-023-40110-6 (PMC10359279; doi:10.1038/s41467-023-40110-6)
Supplement: Supplementary file 1 — Supplementary information [file 41467_2023_40110_MOESM1_ESM.pdf]

Supplementary Information

Experimental Manifestation of Redox-Conductivity in Metal-Organic Frameworks and its Exploitation for Semiconductor/Insulator switching

Jingguo Li,[1#] Amol Kumar,[1#] Ben A. Johnson,[1, 2] Sascha Ott[1]*

[1]Department of Chemistry−Ångström Laboratory, Uppsala University, 75120 Uppsala, Sweden

[2]Technical University of Munich, Campus Straubing for Biotechnology and Sustainability, Uferstraße 53, Straubing 94315, Germany

Table of Contents

1. Theoretical calculation of conductivity in redox-active MOF thin-film 3

2. Notation for the redox state of the MOF thin-film 3

3. Characterization of the pyrazol-NDI linker and Zn(pyrazol-NDI) thin-film 4

4. Experimental EIS data of Zn(pyrazol-NDI) MOF thin-film at different redox states and fittings with equivalent circuits 9

5. Redox switchability and structural stability of Zn(pyrazol-NDI) MOF thin-film.............................. 14

6. Characterization of the dcphOH-NDI linker and Zr(dcphOH-NDI) thin-film 16

7. Experimental EIS data of Zr(dcphOH-NDI) MOF thin-film at different redox states and fittings with equivalent circuits 19

8. Characterization of the UU-100(Co) thin-film 23

9. Experimental EIS data of UU-100(Co) MOF thin-film at different redox states and fittings with equivalent circuits.. 25

10. Experimental EIS data of Zn(pyrazol-NDI) MOF thin-film at different redox states (Li^+ as counterions) and fittings with equivalent circuits 28

11. Experimental EIS data of Zn(pyrazol-NDI) MOF thin-film at different redox states (TBA^+ as counterions) and fittings with equivalent circuits 32

12. Comparison of maximum redox conductivities and corresponding activation barriers of Zn(pyrazol-NDI) MOF thin-films as the function of counterions.. 36

13. References 37

1. Theoretical calculation of conductivity in redox-active MOF thin-film

The calculation of redox conductivity presented in **Fig. 1e** follows previous investigations[1] and the following preconditions are applied: i), defect free MOF thin-film where redox-active linkers are homogeneously distributed and immobile with in MOF matrix; ii), electron injection at the electrode-MOF thin-film interface is fast enough and the redox equilibria of the thin-film obey Nernstian behavior; iii), electron-hopping proceeds with the constrain of electroneutrality, i.e. without consideration of ion-pairing effects; iv), the presence of excess amount of counterions so that the dimensionless conductivity, σ, is primarily depending on the redox equilibria, $\frac{F}{RT}(E - E_0)$. Therefore, electron hopping between immobile redox-active sites is described by a modified Nernst-Planck equation by assuming the validity of the Einstein relationship between the electron mobility and the diffusion coefficient.[2] In the case of a small potential difference between the working and the counter electrodes, the redox conductivity can be estimated by the following equation:

$$\sigma = \frac{F^2 D_e C_0}{RT} \frac{exp\left[\frac{F}{RT}(E-E_0)\right]}{\left\{1+exp\left[\frac{F}{RT}(E-E_0)\right]\right\}^2} \qquad \text{Supplementary Equation 1}$$

Where F is Faraday constant, R is the gas constant, T is the temperature, D_e is the apparent diffusion coefficient, C_0 is the total concentration of redox-active species, E is the applied potential and E_0 is the standard potential of the redox-active species. It is clear that when $E = E_0$, a maximum conductivity will occur, $\sigma_{max} = F^2 D_e C_0 / 4RT$

2. Notation for the redox state of the MOF thin-film

The redox state of the MOF thin-film is directly related to the mole fraction of the follow three species, $[NDI]$, $[NDI^{-\cdot}]$ and $[NDI^{2-}]$, which is fundamentally determined by the applied potential relative to the standard potential of $NDI/NDI^{-\cdot}$ and $NDI^{-\cdot}/NDI^{2-}$ redox couples. For convenience, the redox state of the MOF thin-film can be expressed as the mole fraction of electrons x, where $x = [NDI^{-\cdot}] + 2[NDI^{2-}]$. Therefore, x ranges from 0 to 2 depending on the applied potential, and x equals 0.5 and 1.5 respectively at two standard potentials. In the ideal Nernstian process, the mole fraction of each species can be calculated using following equations:

$$[NDI]_E = \frac{1}{1+exp\left[-\frac{F}{RT}(E-E_1)\right]} \qquad \text{Supplementary Equation 2}$$

$$[NDI^{-\cdot}]_E = \frac{1}{1+exp\left[\frac{F}{RT}(E-E_1)\right]+exp\left[-\frac{F}{RT}(E-E_2)\right]}$$ Supplementary Equation 3

$$[NDI^{2-}]_E = \frac{1}{1+exp\left[\frac{F}{RT}(E-E_2)\right]}$$ Supplementary Equation 4

Where F is Faraday constant, R is the gas constant, T is the temperature, E is the applied potential, E_1 and E_2 are the standard potential of $NDI/NDI^{-\cdot}$ and $NDI^{-\cdot}/NDI^{2-}$ redox couples respectively.

3. Characterization of the pyrazol-NDI linker and Zn(pyrazol-NDI) thin-film

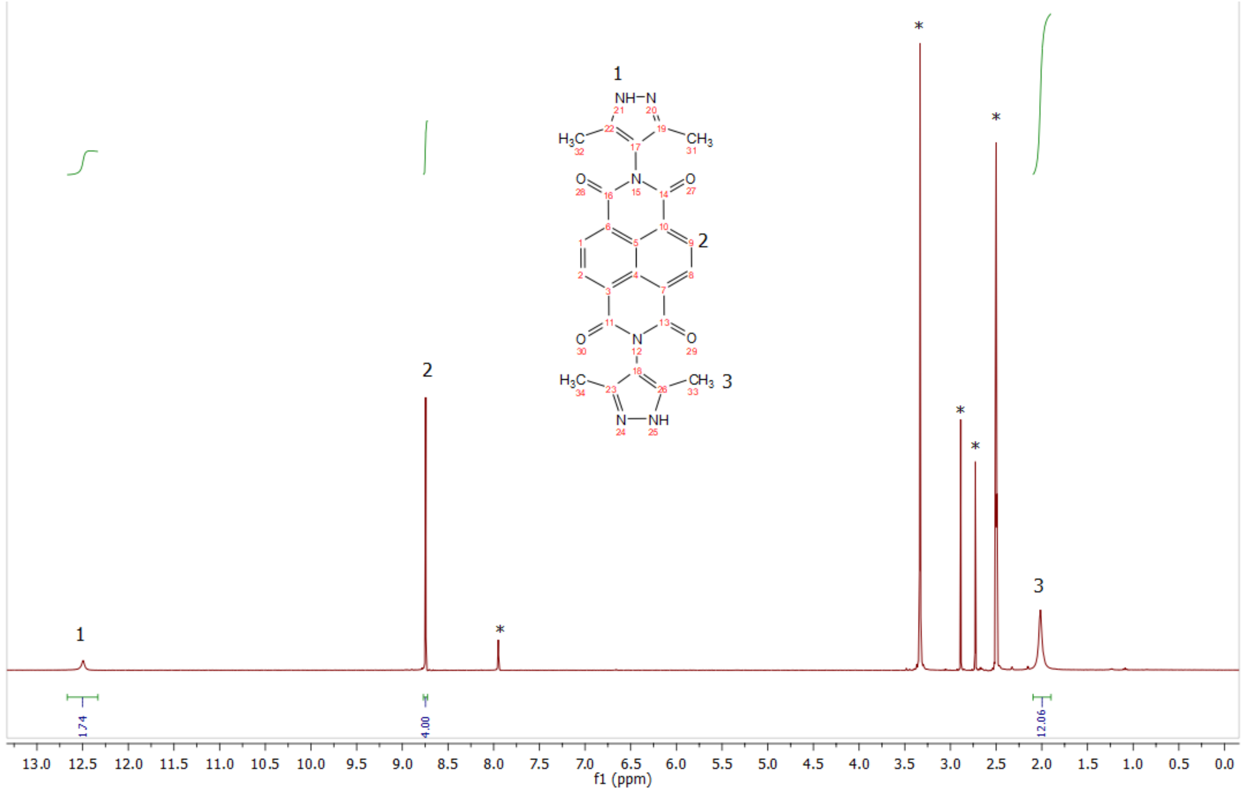

Supplementary Figure 1. ^{1}HNMR of pyrazol-NDI linker measured in DMSO-d_6 at 293 K. The nondeuterated solvent and water are marked with asterisk sign.

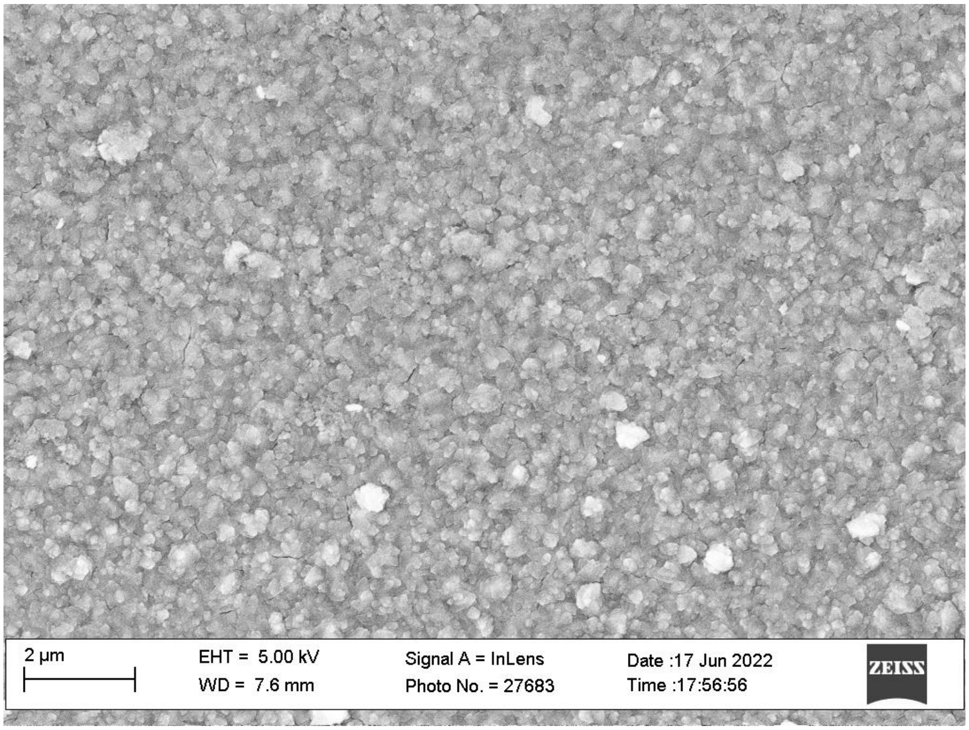

Supplementary Figure 2. SEM top surface image of the Zn(pyrazol-NDI) thin-film. The thin-film is compact and uniformly grown on the surface of FTO substrate.

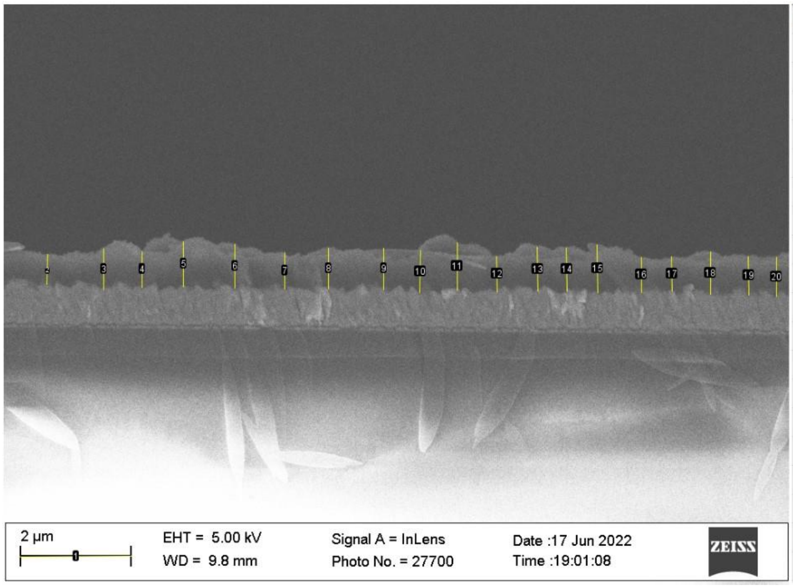

Measurements	Length (µm)
1	0.54
2	0.73
3	0.63
4	0.8
5	0.79
6	0.65
7	0.73
8	0.74
9	0.76
10	0.86
11	0.63
12	0.79
13	0.77
14	0.86
15	0.63
16	0.62
17	0.76
18	0.69
19	0.7

Supplementary Figure 3. SEM cross-section image of the Zn(pyrazol-NDI) thin-film on FTO surface and the measurements of film thickness were done across image using ImageJ program and labelled in yellow bars (left). The measurement results were listed in the right.

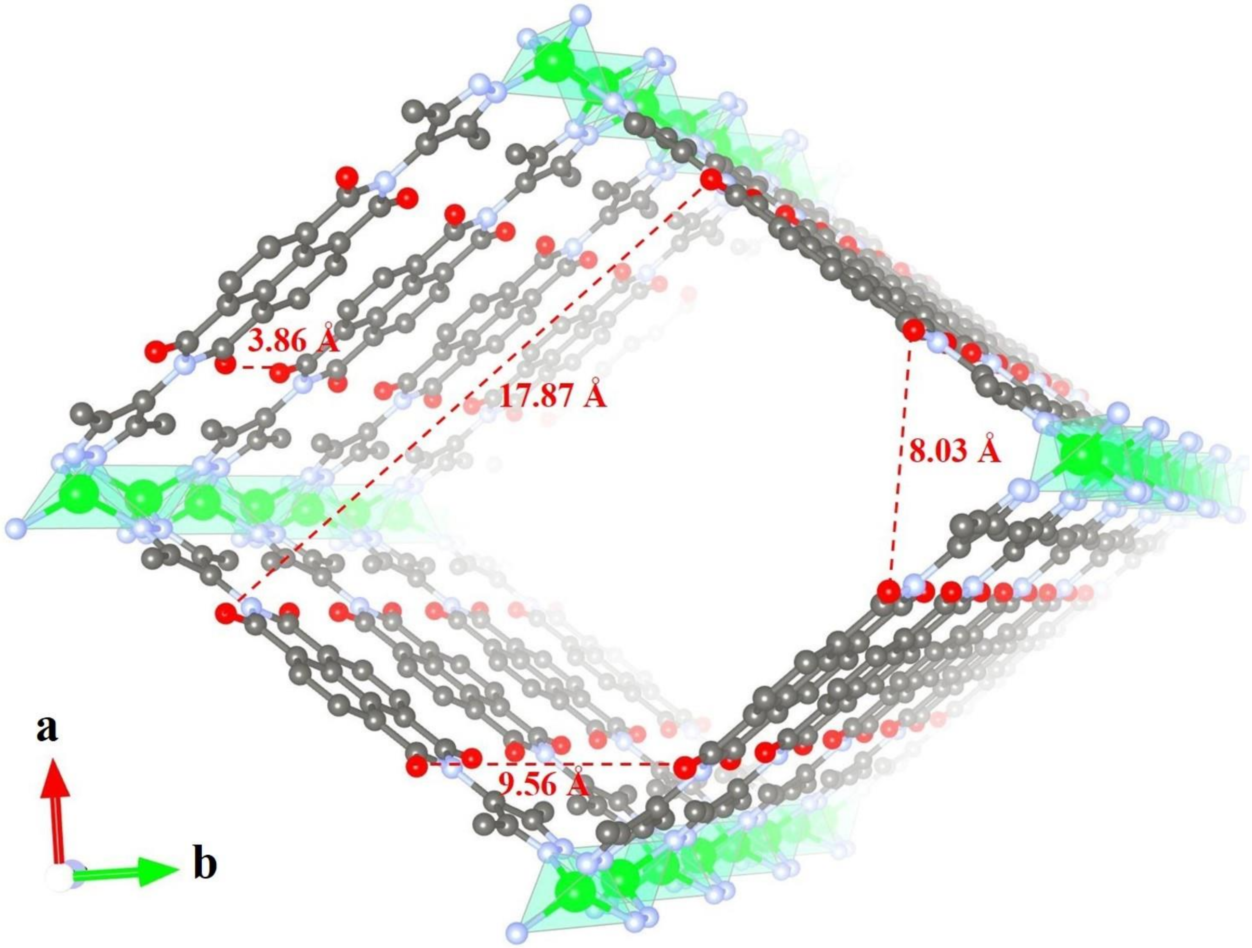

Supplementary Figure 4. The crystal structure of Zn(pyrazol-NDI) highlighting the effective intermolecular distance for redox-hopping. Hydrogen atoms are omitted for clarity; C, N, O, and Zn atoms or ions are presented with gray, blue, red, and green spheres, respectively (the crystal structure was reconstructed based on the reported structure in ref.[3]).

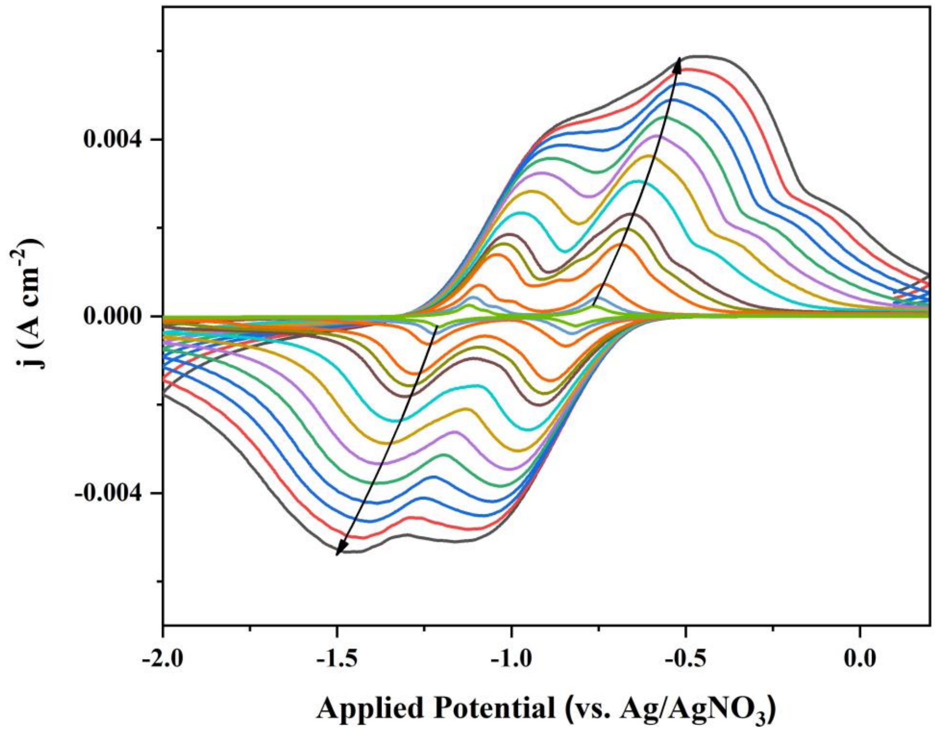

Supplementary Figure 5. Scan-rate-dependent CVs of Zn(pyrazol-NDI) thin-film on FTO at scan rates from 5 to 500 mV s^{-1} in DMF with 0.1 M KPF_6 as the supporting electrolyte. The arrows indicate the growth of peak current densities as the result of scan-rate increase.

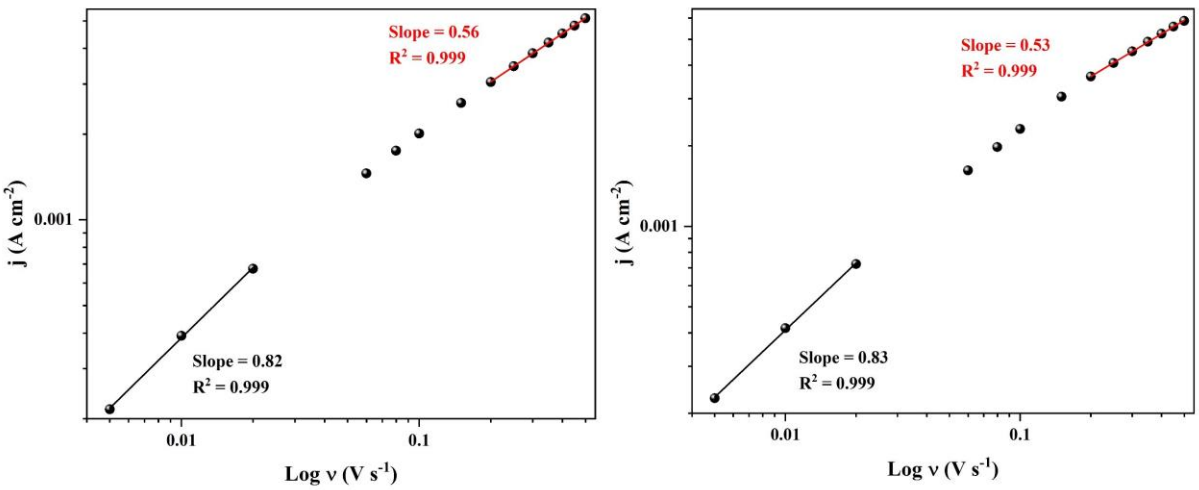

Supplementary Figure 6. Double logarithmic plot for the first cathodic reduction peak current densities (left) and its corresponding re-oxidation peak current densities (right) during anodic sweep versus different scan rates shown in **Supplementary Figure 5**. Experimental slopes are in line with theoretically predicted slopes of 1 (surface-confined process) and 0.5 (diffusion-controlled process).

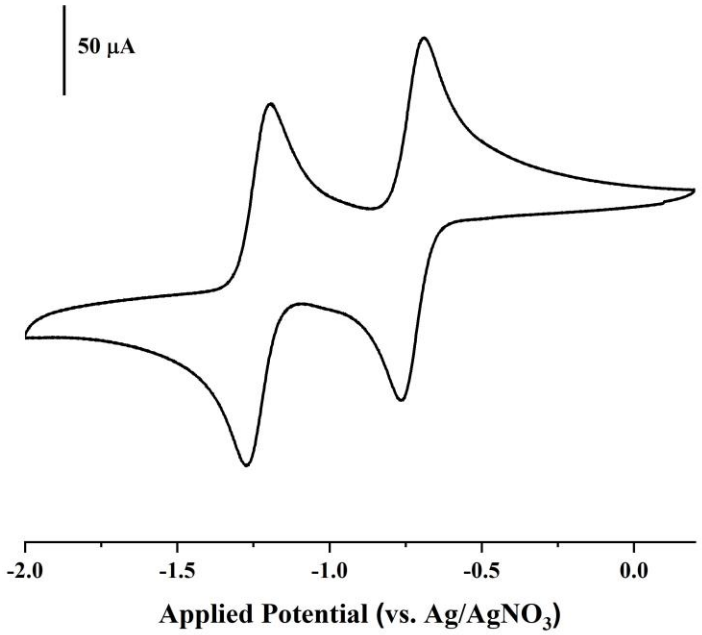

Supplementary Figure 7. CV of pyrazol-NDI linkers measured at scan rates from 50 mV s^{-1} in DMF with 0.1 M KPF_6 as the supporting electrolyte.

4. Experimental EIS data of Zn(pyrazol-NDI) MOF thin-film at different redox states and fittings with equivalent circuits

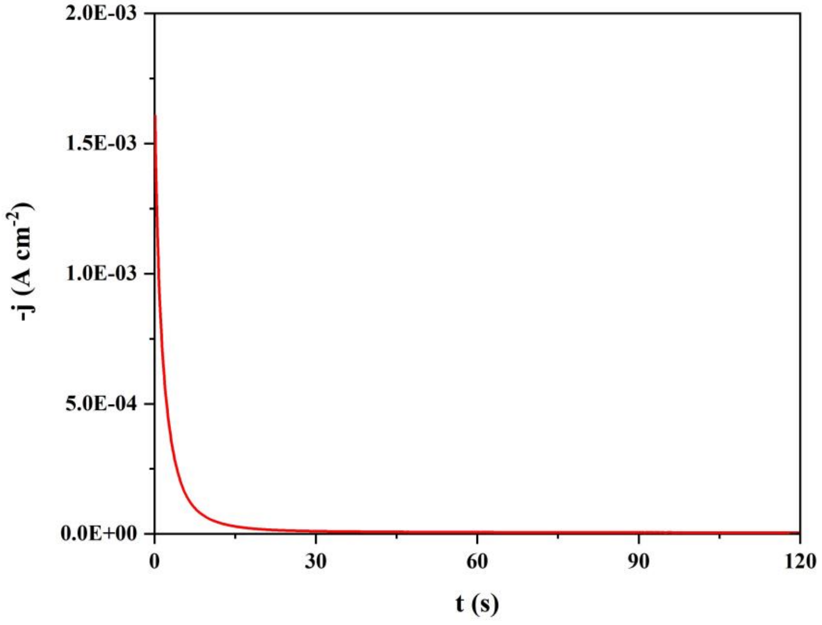

Supplementary Figure 8. An exemplary chronoamperometry of the Zn(pyrazol-NDI) thin-film in DMF (0.1 M KPF_6 as the supporting electrolyte) with a potential step from 0 to -0.78 V, lasting a period of 120 s to reach steady-state for the following impedance measurements.

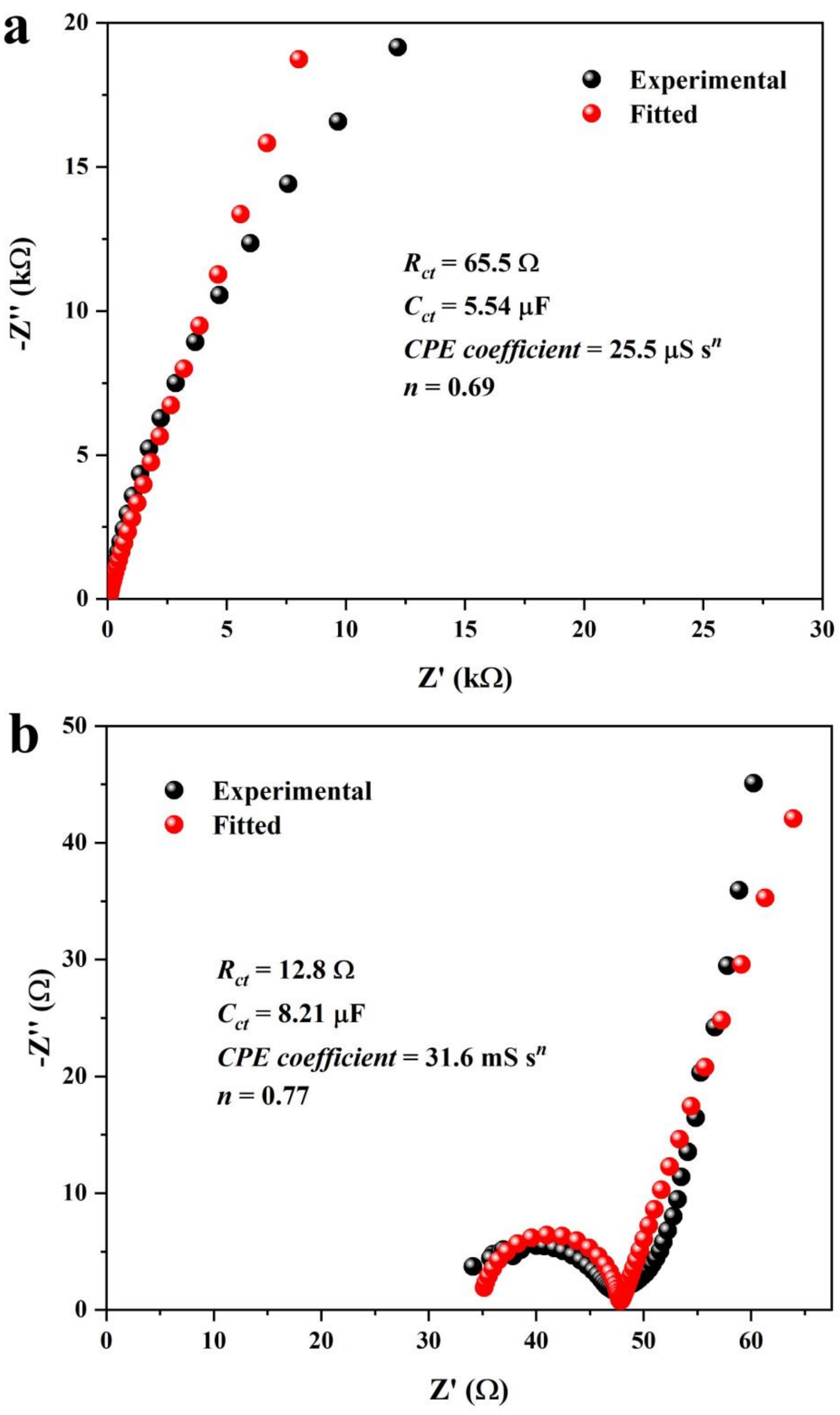

Supplementary Figure 9. Nyquist plots of experimental and fitted (circuit in **Supplementary Figure 11b**) electrochemical impedance data for *0.0*-Zn(pyrazol-NDI) (a) and *0.5*-Zn(pyrazol-NDI) (b) thin-films measured in 0.1 M KPF_6 DMF electrolyte.

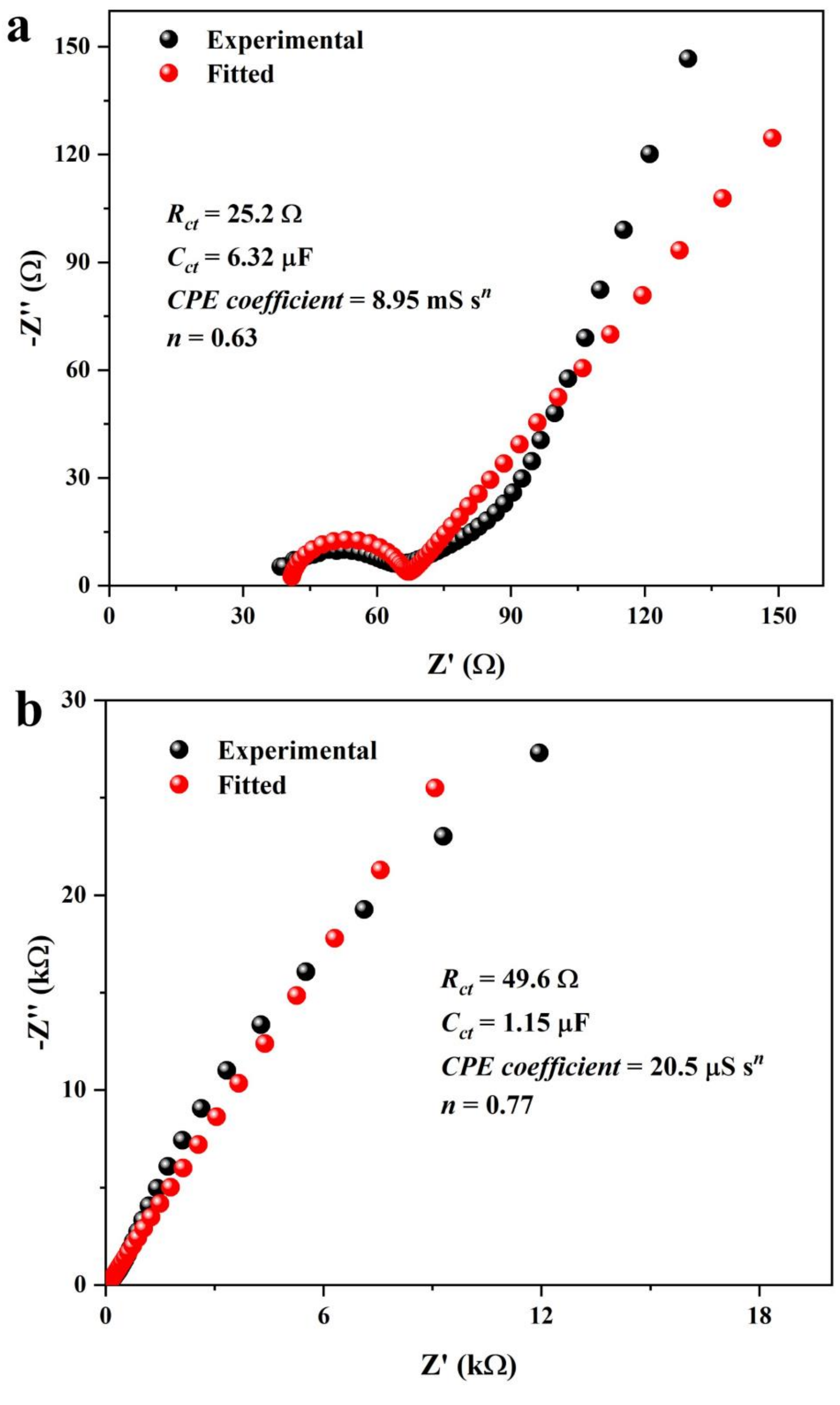

Supplementary Figure 10. Nyquist plots of experimental and fitted (circuit in **Supplementary Figure 11b**) electrochemical impedance data for *1.5*-Zn(pyrazol-NDI) (a) and *2.0*-Zn(pyrazol-NDI) (b) thin-films measured in 0.1 M KPF_6 DMF electrolyte.

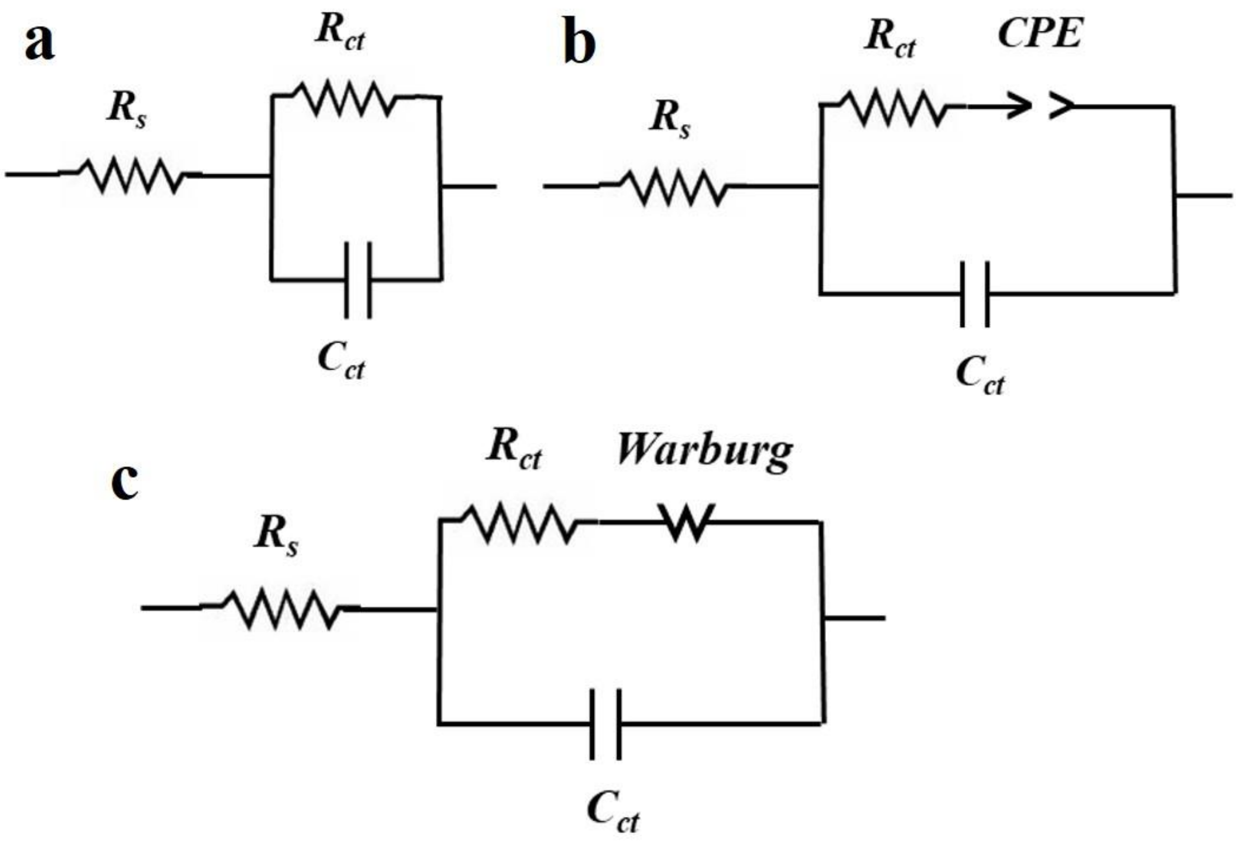

Supplementary Figure 11. Equivalent circuits (EC) for simulation of the experimental impedance date. (a) Simplified RC circuit, where R_{ct} and C_{ct} stand for resistance and capacitance related to the inter-site cation coupled electron hopping. (b) Modified RC circuit by adding the serial a constant phase element (CPE) to the electronic resistance component, where CPE is primarily defined by its phase, n ($0 \leq n \leq 1$), when n equals 1, 0.5, or 0, the CPE represent an ideal capacitor, a semi-infinite diffusional Warburg element, and an ideal resistor, respectively. (c) Modified RC circuit by adding the serial Warburg element to the electronic resistance component. In all circuits, Rs stands for the resistance related to electrolyte.

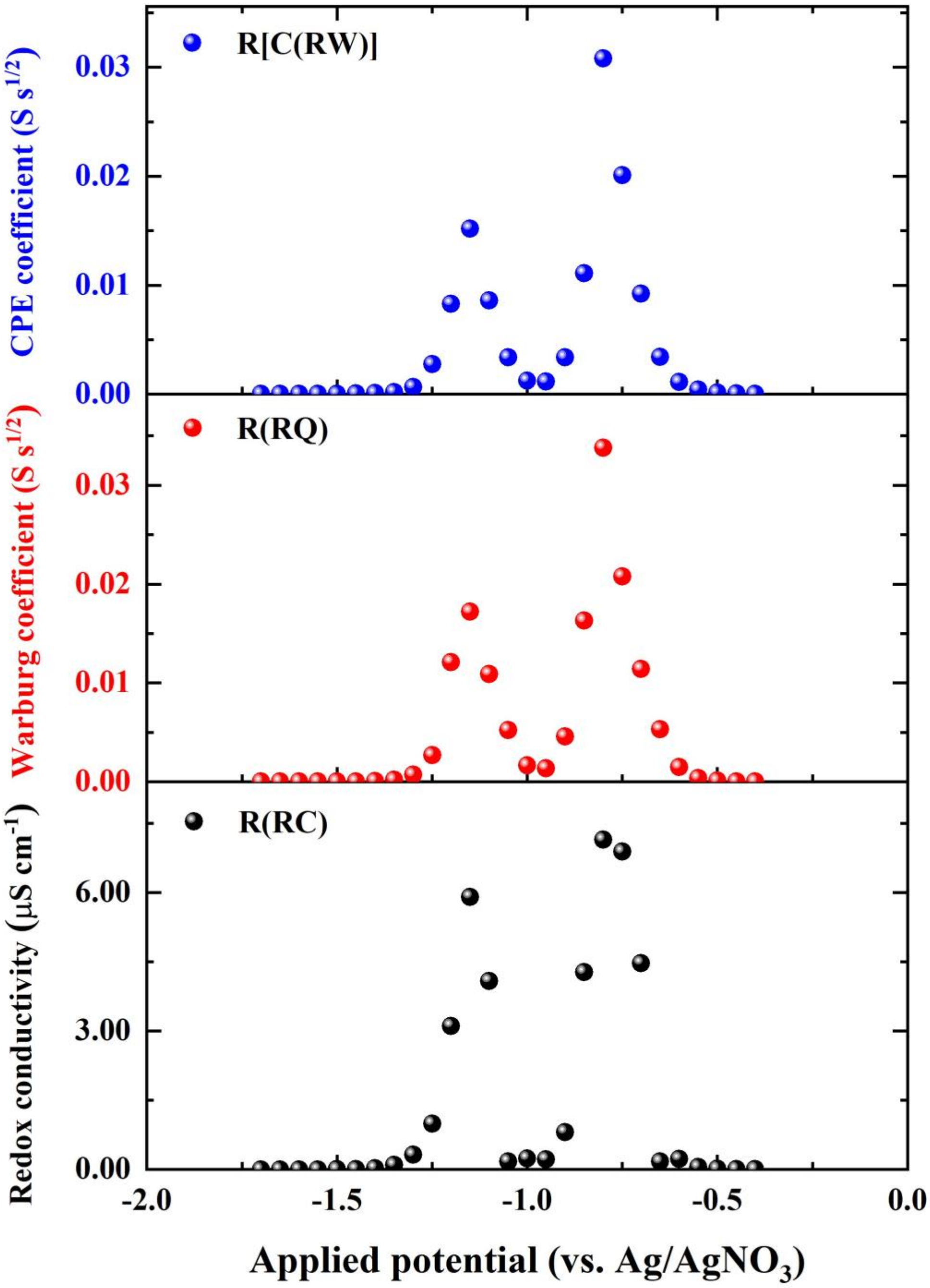

Supplementary Figure 12. Evolution of redox conductivity (lower panel, simulated from **Supplementary Figure 11a**), Warburg coefficient (middle panel, simulated from **Supplementary Figure 11c**) and CPE coefficient (upper panel, simulated from **Supplementary Figure 11b**) as the function of electrochemical potential (redox state of the thin-film) measured in 0.1 M KPF_6 DMF electrolyte, which is determined by the mole fraction of electron reduction, *x*-Zn(pyrazol-NDI), $0.0 \leq x \leq 2.0$. Note that the unit of the Y-axes for upper panel depends upon the phase, *n*; to facilitate comparison, *n* was assumed to be 0.5 here.

The consistent evolution feature of the redox conductivity fitted from different equivalent circuits suggests that electron hopping is most probably coupled with counterion movement during the AC conductivity measurement. For such a coupled process, physical models which emphasize either electrical resistance element (**Supplementary Figure 11a**) or ionic semi-infinite diffusional element (**Supplementary Figure 11c**) as the rate limiting process cannot fully interpret this ion-coupled electron hopping nature. Using a constant phase element in **Supplementary Figure 11b** significantly increases the complexity of the model, while it also delivers better fittings for experimental data. To facilitate easy comparison and discussions of the redox conductivity, we presented the fitted result using the equivalent circuit shown in **Supplementary Figure 11a** in the main text.

5. Redox switchability and structural stability of Zn(pyrazol-NDI) MOF thin-film

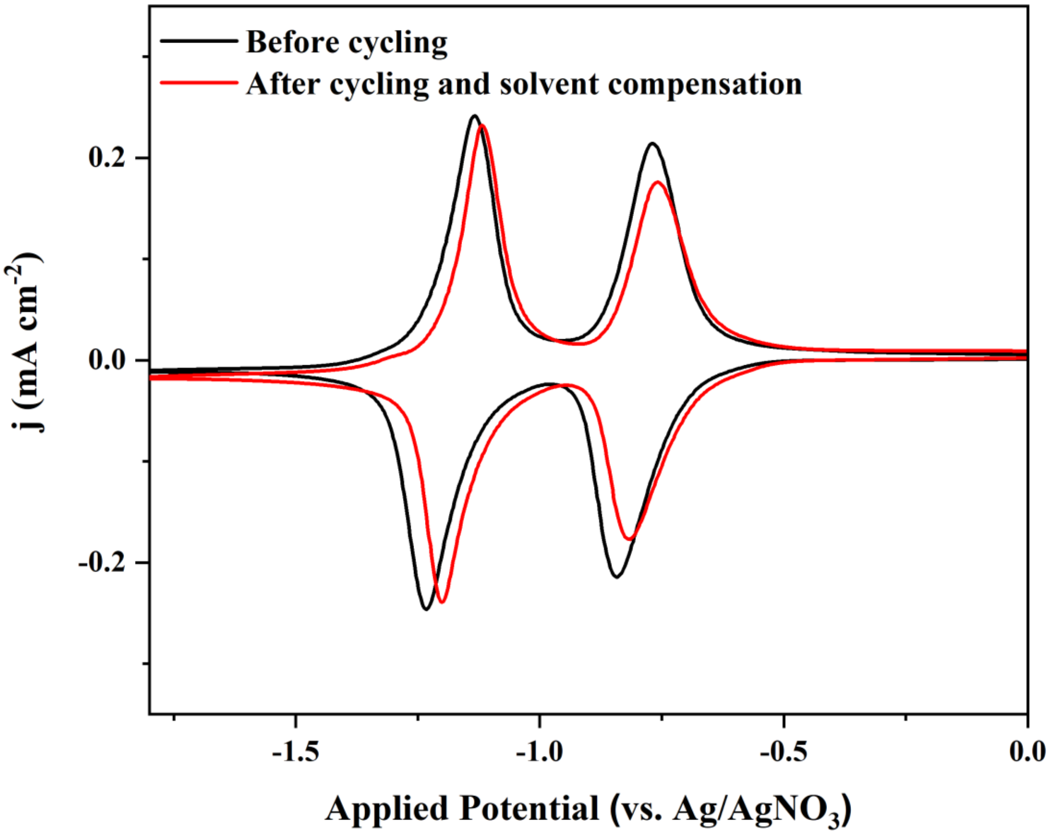

Supplementary Figure 13. CV (10 mV s^{-1}) change of MOF thin-film before (black) and after (red, solvent compensated) redox conductivity cycling between x = 0.0 and x = 0.5 over 100 cycles (~ 24 h operation). Both redox waves shifted anodically ~ 20 mV, the loss of MOF thin-film is estimated to be ~ 15 % according to the first reduction peak.

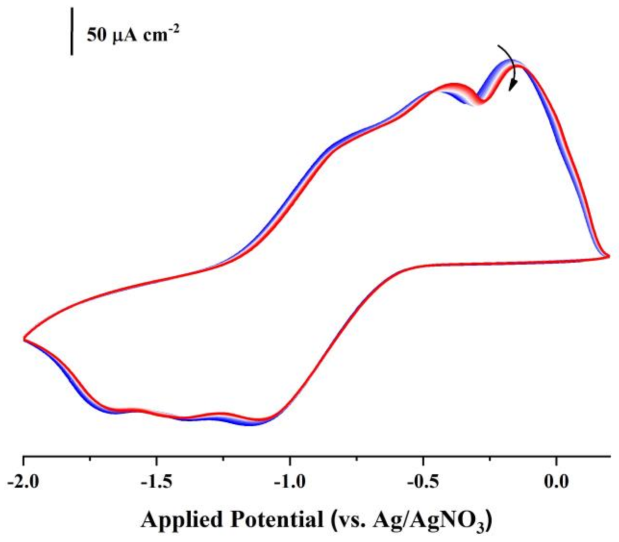

Supplementary Figure 14. 100-CV cycles of a fresh-made Zn(pyrazol-NDI) thin-film at scan rates of 100 mV s^{-1} in DMF with 0.1 M KPF_6 as the supporting electrolyte, for easy visualization, only every 10th curves were shown. The arrow indicates the decrease of the peak current density as scan number increases, only ~2.7 % loss is observed after 100 cycles of CV.

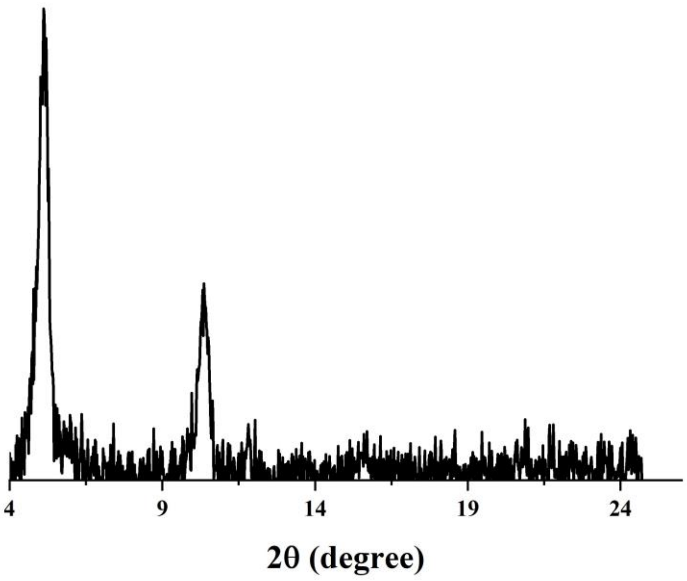

Supplementary Figure 15. Experimental thin-film XRD of Zn(pyrazol-NDI) on FTO surface after 100-CV cycles stability test (see **Supplementary Figure 14**) in DMF with 0.1 M KPF_6 as the supporting electrolyte.

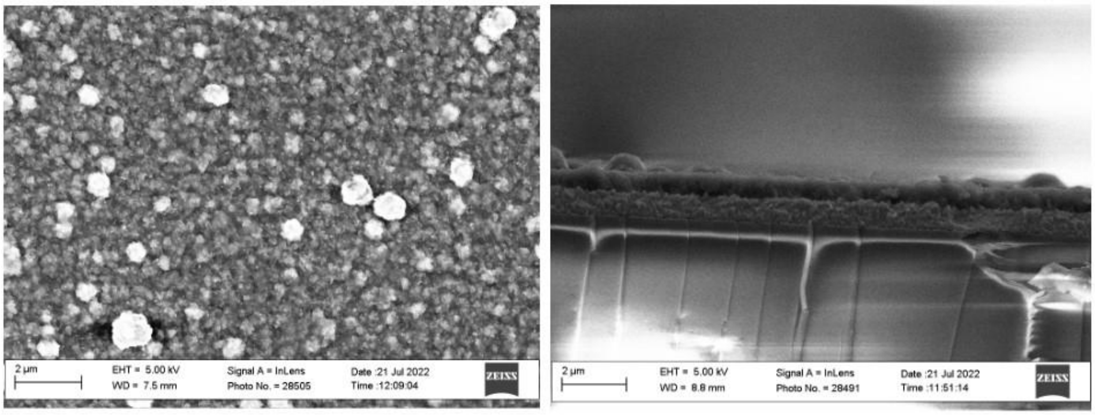

Supplementary Figure 16. SEM top surface (left) and cross-section (right) images of the Zn(pyrazol-NDI) thin-film after 100-CV cycles stability test (see **Supplementary Figure 14**) in DMF with 0.1 M KPF_6 as the supporting electrolyte.

6. Characterization of the dcphOH-NDI linker and Zr(dcphOH-NDI) thin-film

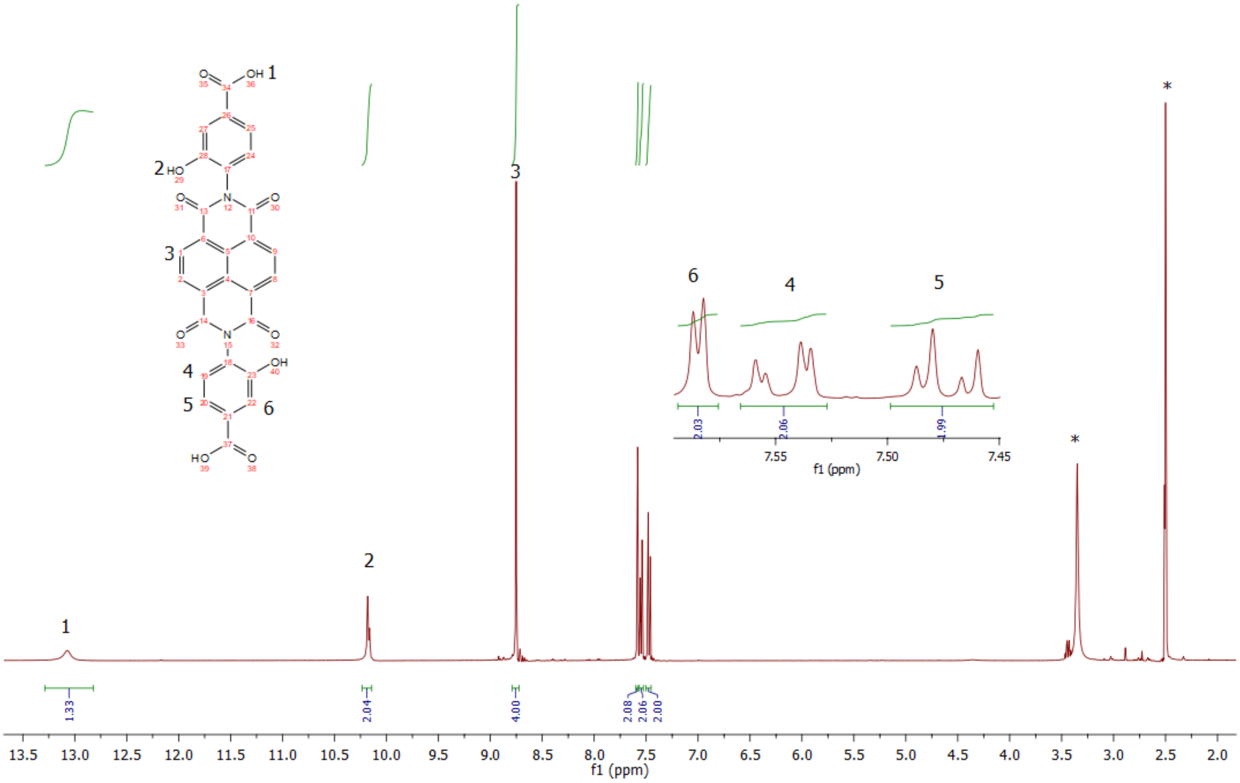

Supplementary Figure 17. 1HNMR of dcphOH-NDI linker measured in DMSO-d_6 at 293 K. The nondeuterated solvent and water are marked with asterisk sign.

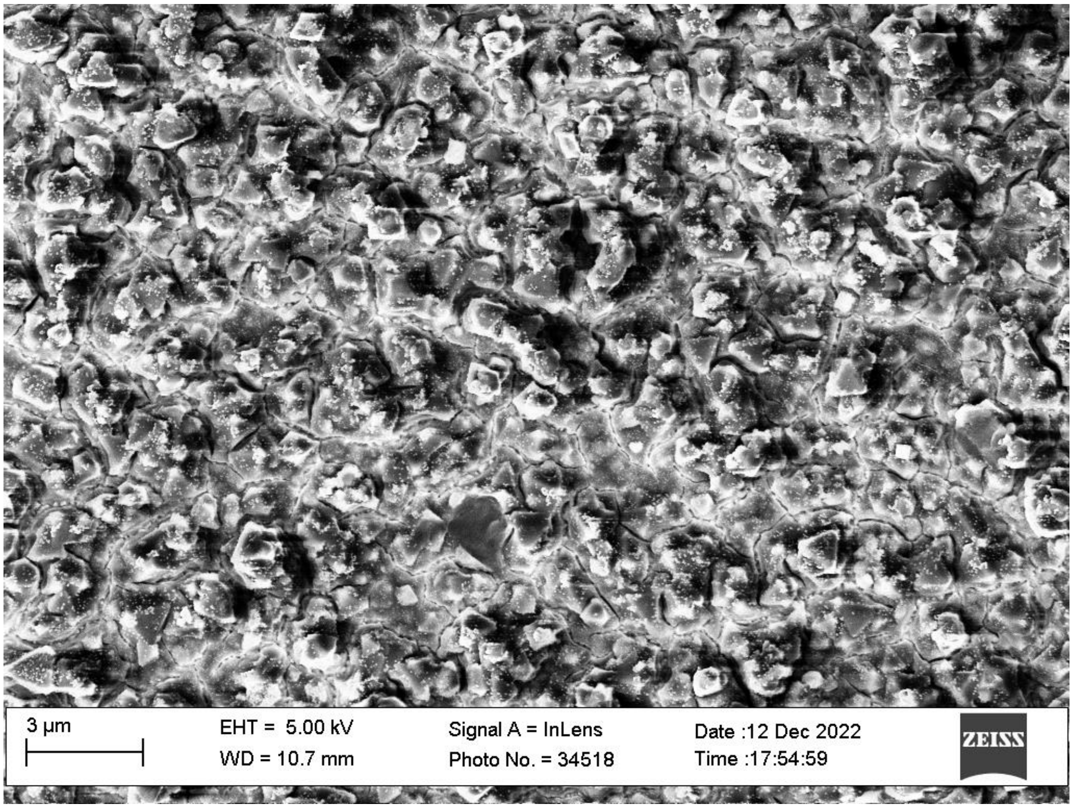

Supplementary Figure 18. SEM top surface image of the Zr(dcphOH-NDI) thin-film. The thin-film is compact and uniformly grown on the surface of FTO substrate.

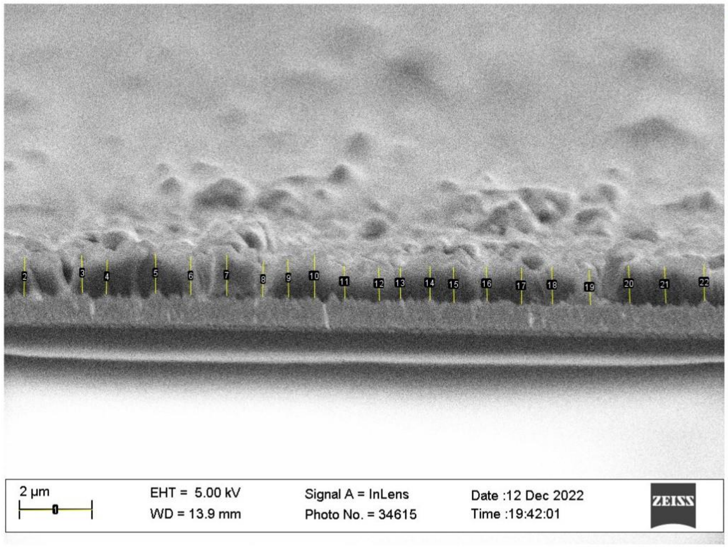

Measurements	Length (µm)
1	1.06
2	1
3	0.92
4	1.06
5	1.02
6	1.1
7	0.94
8	1.02
9	1.15
10	0.88
11	0.88
12	0.83
13	0.9
14	0.96
15	0.9
16	0.98
17	1
18	0.94
19	1
20	0.98
21	0.96

Supplementary Figure 19. SEM cross-section image of the Zr(dcphOH-NDI) thin-film on FTO surface and the measurements of film thickness were done across image using ImageJ program and labelled in yellow bars (left). The measurement results were listed in the right.

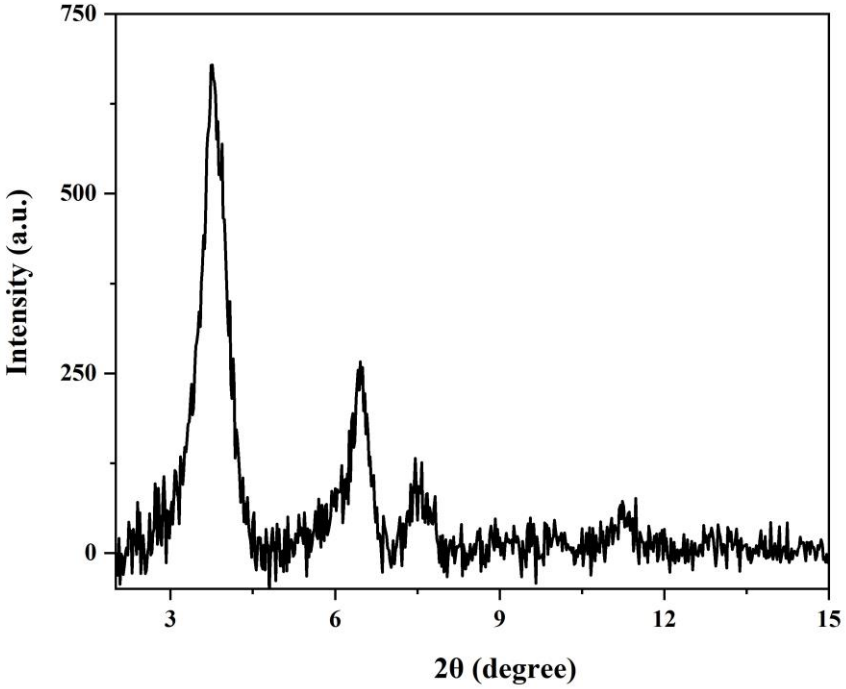

Supplementary Figure 20. Experimental Zr(dcphOH-NDI) thin-film XRD data on FTO surfaces.

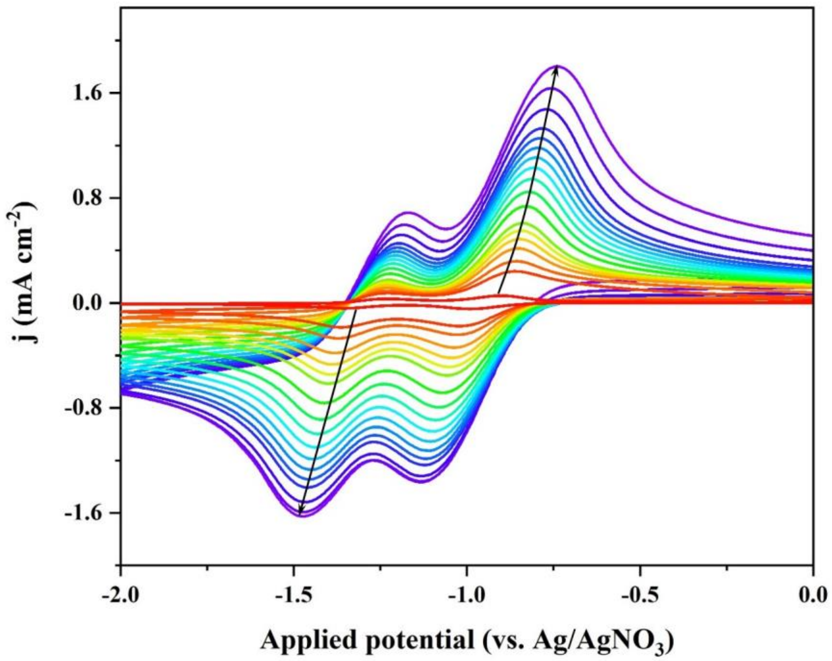

Supplementary Figure 21. Scan-rate-dependent CVs of Zr(dcphOH-NDI) thin-film on FTO at scan rates from 1 to 800 mV s^{-1} in DMF with 0.1 M KPF_6 as the supporting electrolyte. The arrows indicate the growth of peak current densities as the result of scan-rate increase.

7. **Experimental EIS data of Zr(dcphOH-NDI) MOF thin-film at different redox states and fittings with equivalent circuits**

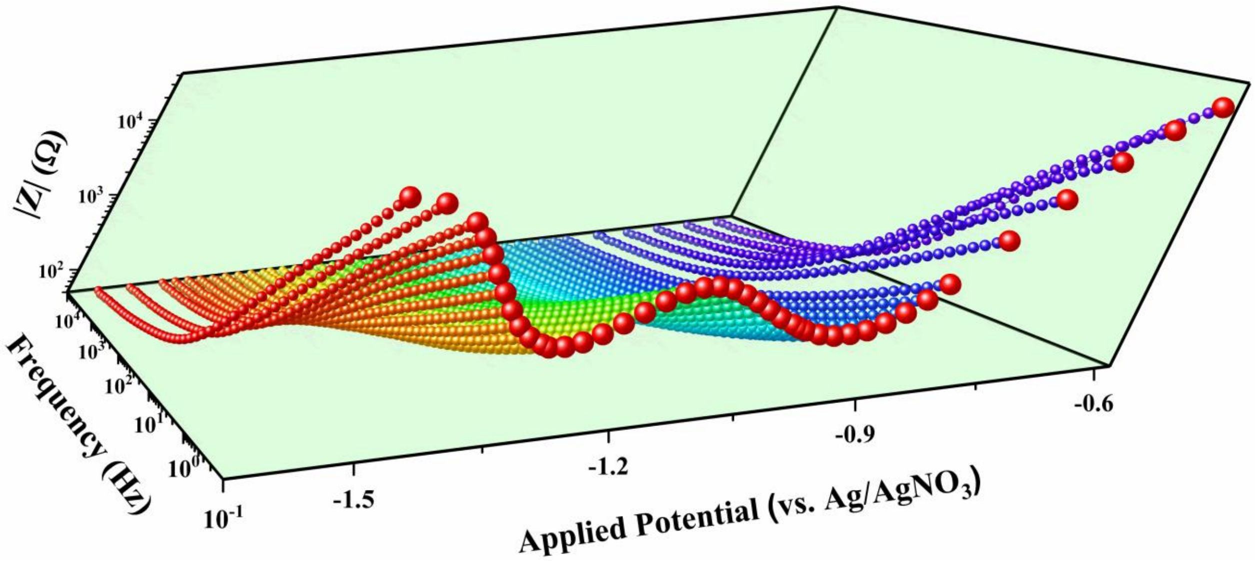

Supplementary Figure 22. Bode plots of Zr(dcphOH-NDI) thin-film at different applied potentials measured in 0.1 M KPF_6 DMF electrolyte (the impedance data point at the frequency of 0.1 Hz was magnified for each measurement to highlight the effect of redox state), before each measurement, a stabilization time of 120 s was applied at respective potentials to achieve steady redox states.

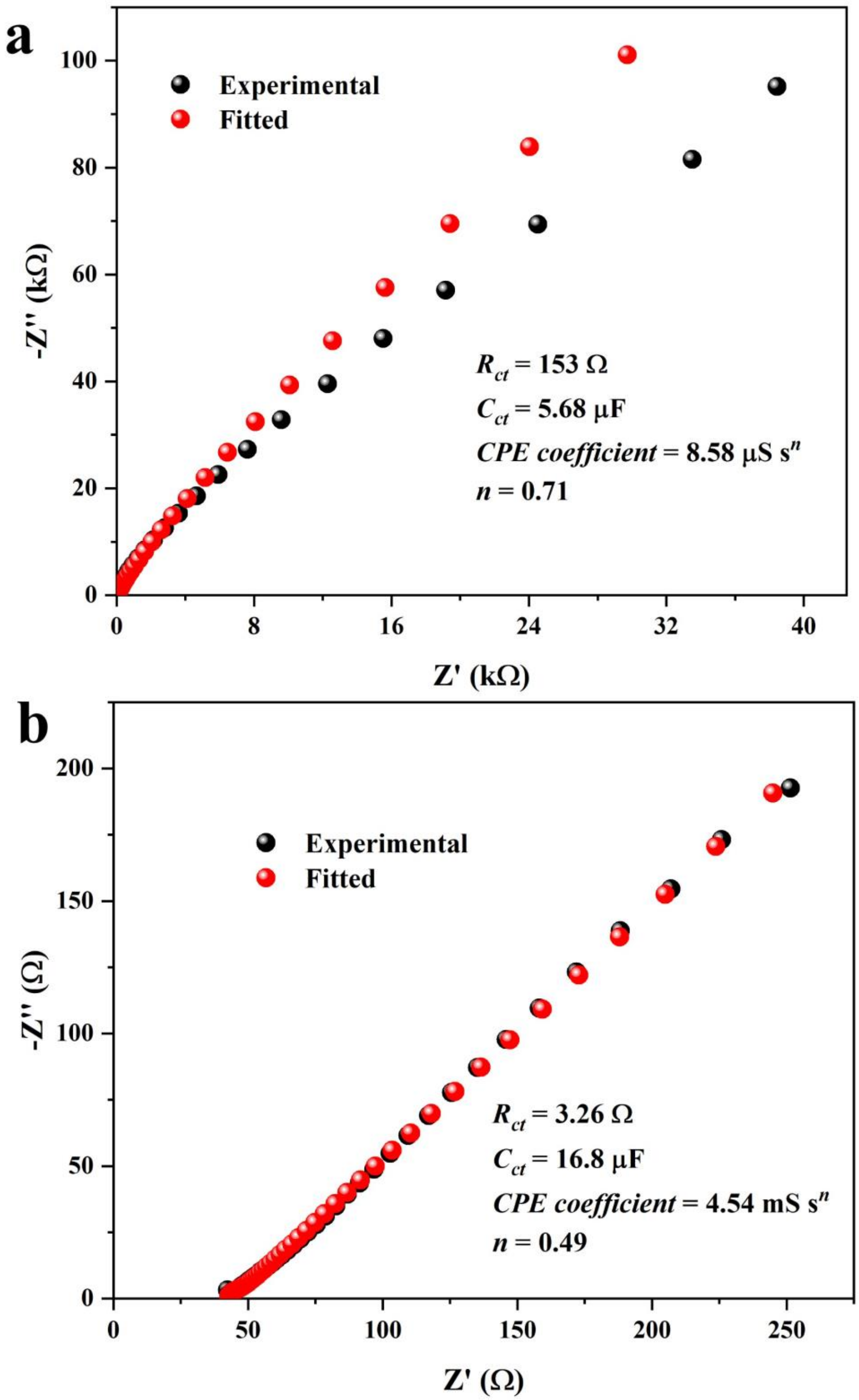

Supplementary Figure 23. Nyquist plots of experimental and fitted (circuit in **Supplementary Figure 11b**) electrochemical impedance data for *0.0*- Zr(dcphOH-NDI) (a) and *0.5*- Zr(dcphOH-NDI) (b) thin-films measured in 0.1 M KPF_6 DMF electrolyte.

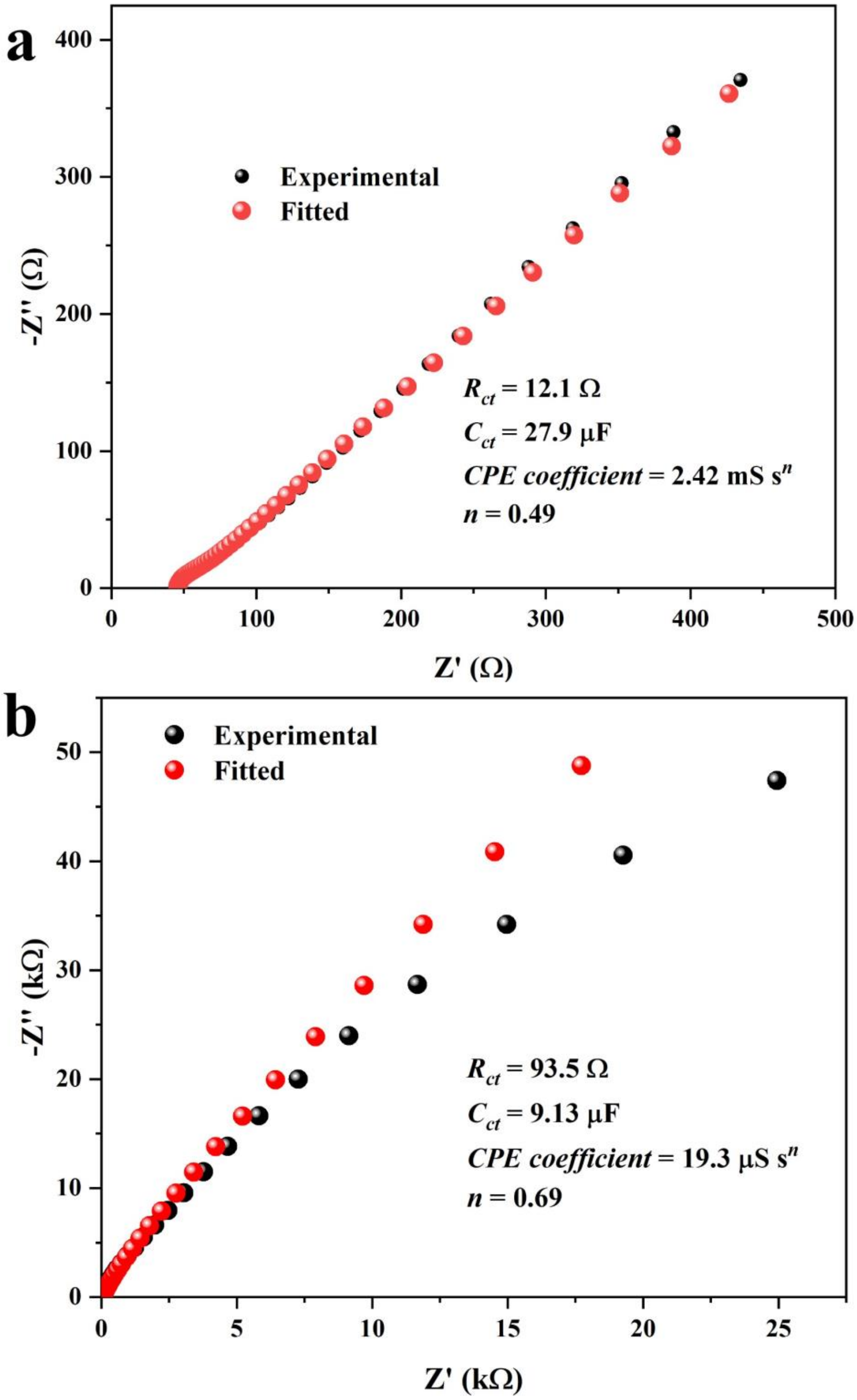

Supplementary Figure 24. Nyquist plots of experimental and fitted (circuit in **Supplementary Figure 11b**) electrochemical impedance data for *1.5*-Zr(dcphOH-NDI) (a) and *2.0*-Zr(dcphOH-NDI) (b) thin-films measured in 0.1 M KPF_6 DMF electrolyte.

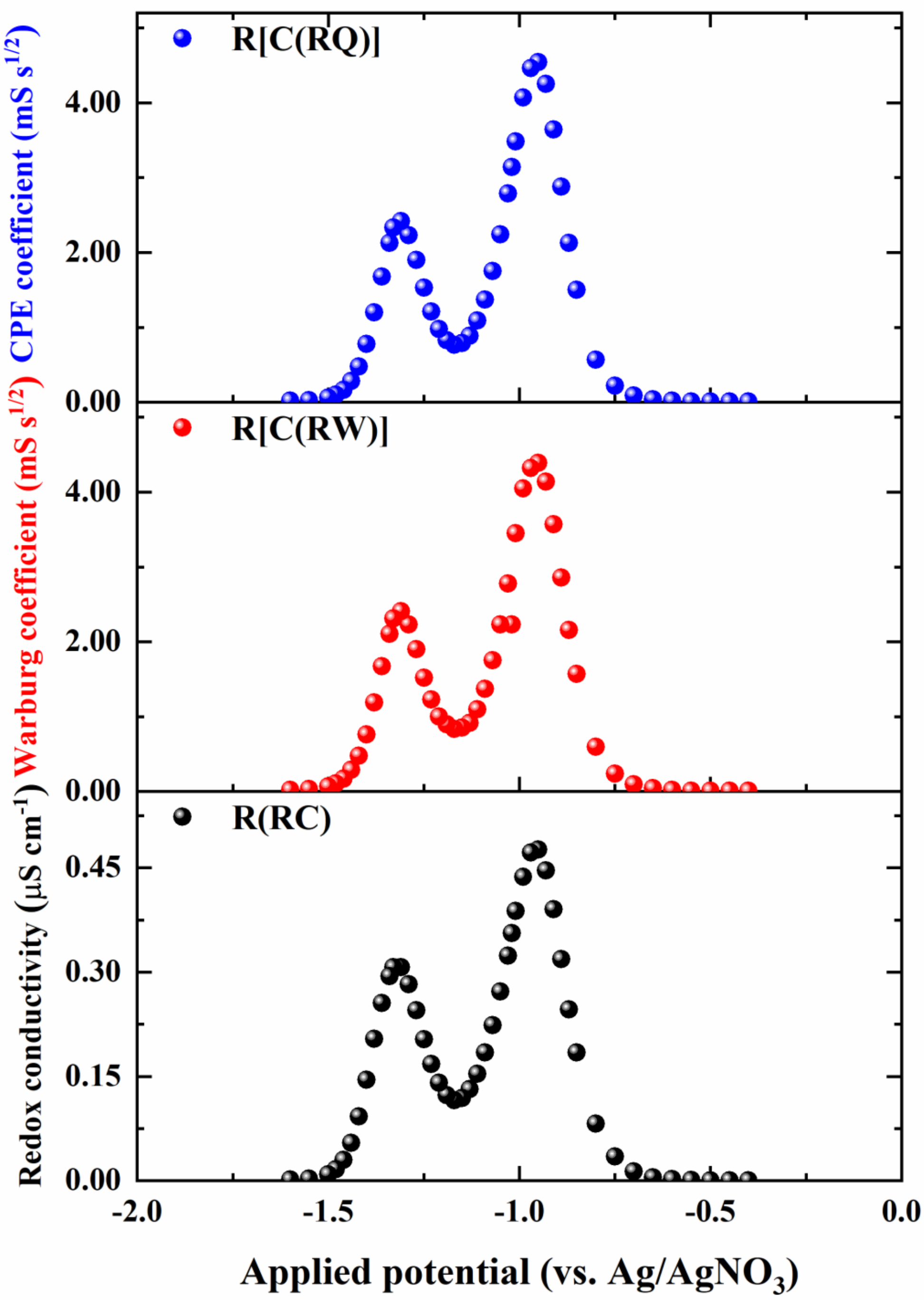

Supplementary Figure 25. Evolution of redox conductivity (lower panel, simulated from **Supplementary Figure 11a**), Warburg coefficient (middle panel, simulated from **Supplementary Figure 11c**) and CPE coefficient (upper panel, simulated from **Supplementary Figure 11b**) as the function of electrochemical potential (redox state of the thin-film) measured in 0.1 M KPF_6 DMF electrolyte, which is determined by the mole fraction of electron reduction, *x*-Zr(dcphOH-NDI), $0.0 \leq x \leq 2.0$. Note that the unit of the Y-axes for upper panel is changing upon the phase, *n*, to facilitate comparison, *n* was assumed to be 0.5 here.

8. Characterization of the UU-100(Co) thin-film

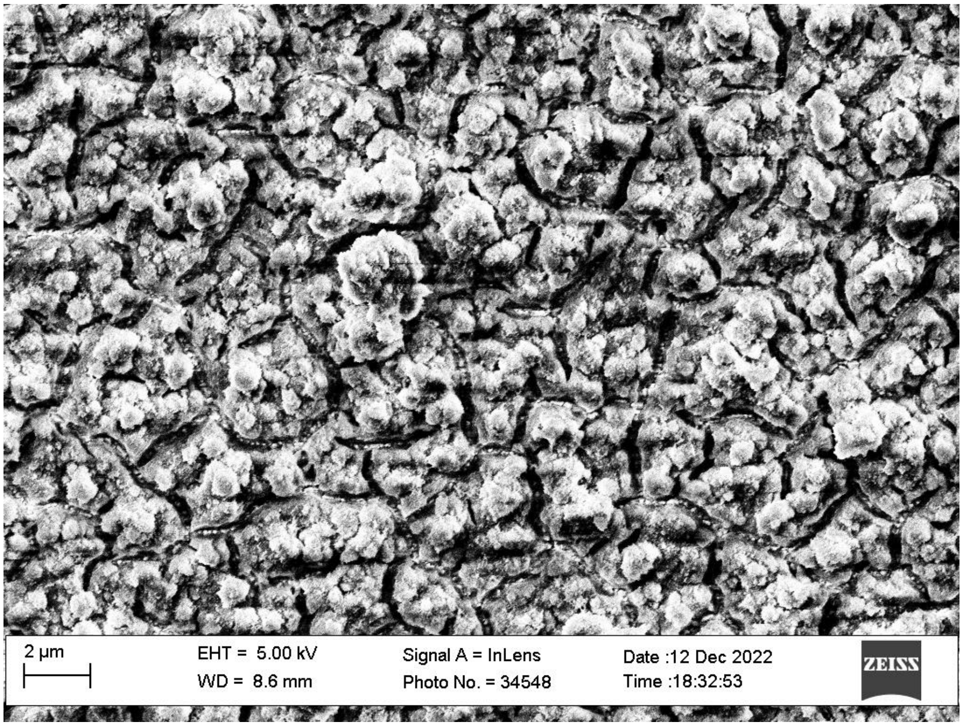

Supplementary Figure 26. SEM top surface image of the UU-100(Co) thin-film. The thin-film is compact and uniformly grown on the surface of FTO substrate.

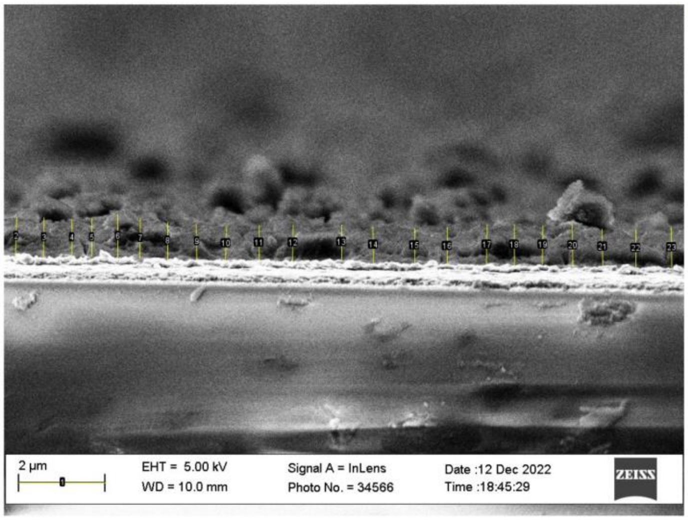

Measurements	Length (µm)
1	0.9
2	0.93
3	0.85
4	0.9
5	1.02
6	0.94
7	0.86
8	0.82
9	0.8
10	0.82
11	0.87
12	0.82
13	0.83
14	0.82
15	0.87
16	0.93
17	0.98
18	0.91
19	1
20	0.85
21	0.87
22	0.96

Supplementary Figure 27. SEM cross-section image of the UU-100(Co) thin-film on FTO surface and the measurements of film thickness were done across image using ImageJ program and labelled in yellow bars (left). The measurement results were listed in the right.

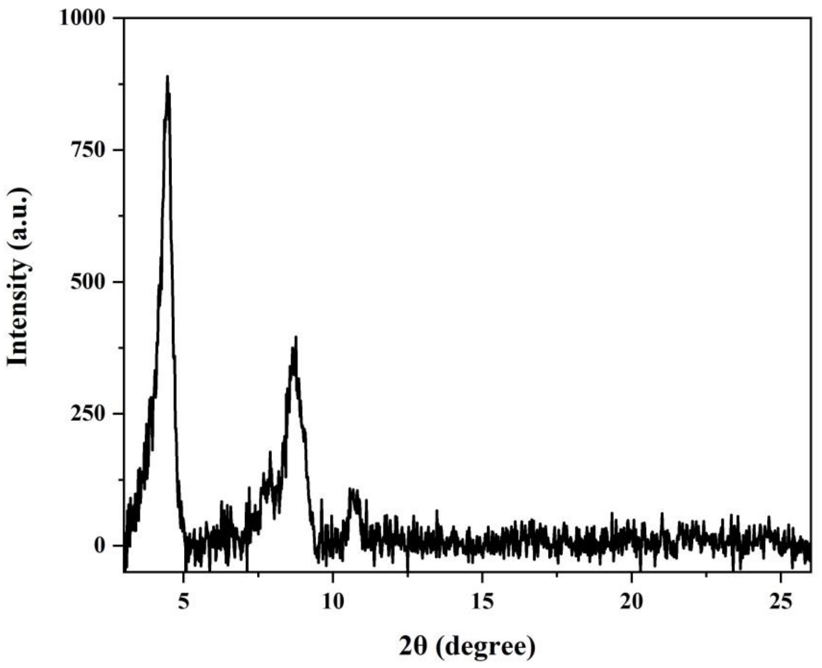

Supplementary Figure 28. Experimental UU-100(Co) thin-film XRD data on FTO surfaces.

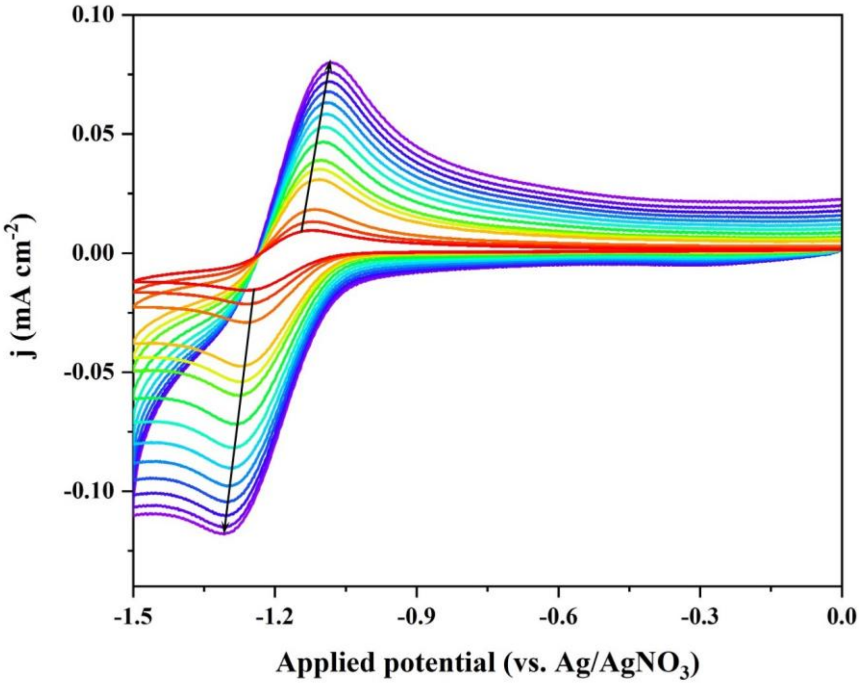

Supplementary Figure 29. Scan-rate-dependent CVs of UU-100(Co) thin-film on FTO at scan rates from 5 to 500 mV s^{-1} in DMF with 0.1 M KPF_6 as the supporting electrolyte. The arrows indicate the growth of peak current densities as the result of scan-rate increase.

9. Experimental EIS data of UU-100(Co) MOF thin-film at different redox states and fittings with equivalent circuits

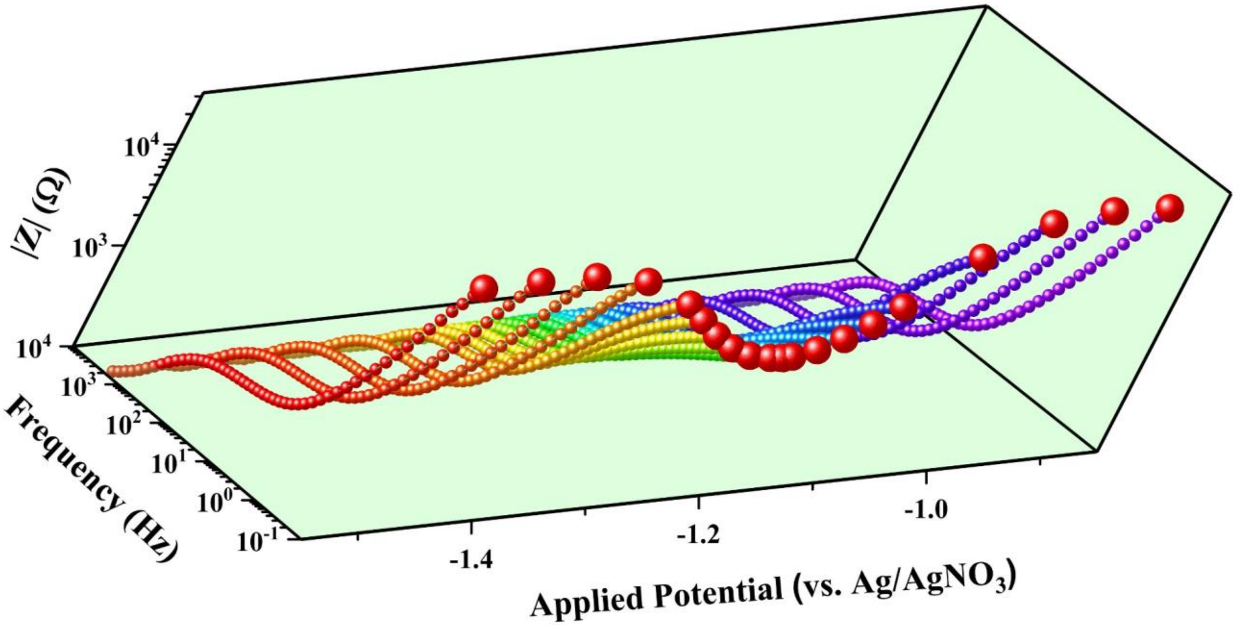

Supplementary Figure 30. Bode plots of UU-100(Co) thin-film at different applied potentials measured in 0.1 M KPF_6 DMF electrolyte (the impedance data point at the frequency of 0.1 Hz was magnified for each measurement to highlight the effect of redox state), before each measurement, a stabilization time of 120 s was applied at respective potentials to achieve steady redox states.

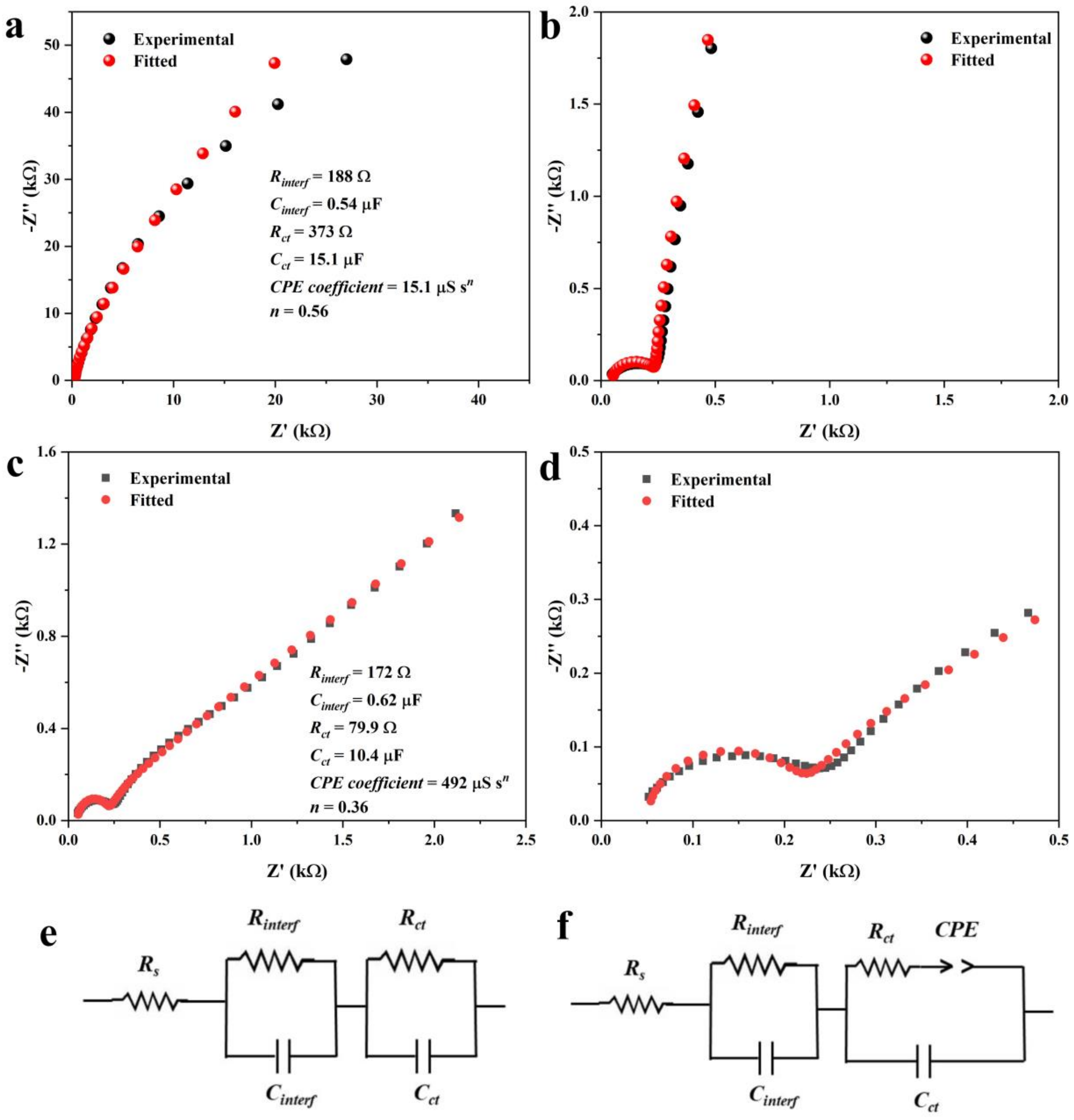

Supplementary Figure 31. Nyquist plots of experimental and fitted (circuit in **f**) electrochemical impedance data for *0.0*-UU-100(Co) (a-b), *0.5*-UU-100(Co) (c-d), thin-films measured in 0.1 M KPF_6 DMF electrolyte. Since a consistent high frequency semicircle was observed for all measurements for UU-100(Co) films, which can be assign to the interfacial charge transfer between FTO surface and MOF thin-film. Therefore, two RC circuits (e) are employed to simulate our experiment data. (f) Modified RC circuit by adding the serial a constant phase element (CPE) to the electronic resistance component, where CPE is primarily defined by its phase, n ($0 \leq n \leq 1$), when n equals 1, 0.5, or 0, the CPE represent an ideal capacitor, a semi-infinite diffusional Warburg element, and an ideal resistor, respectively.

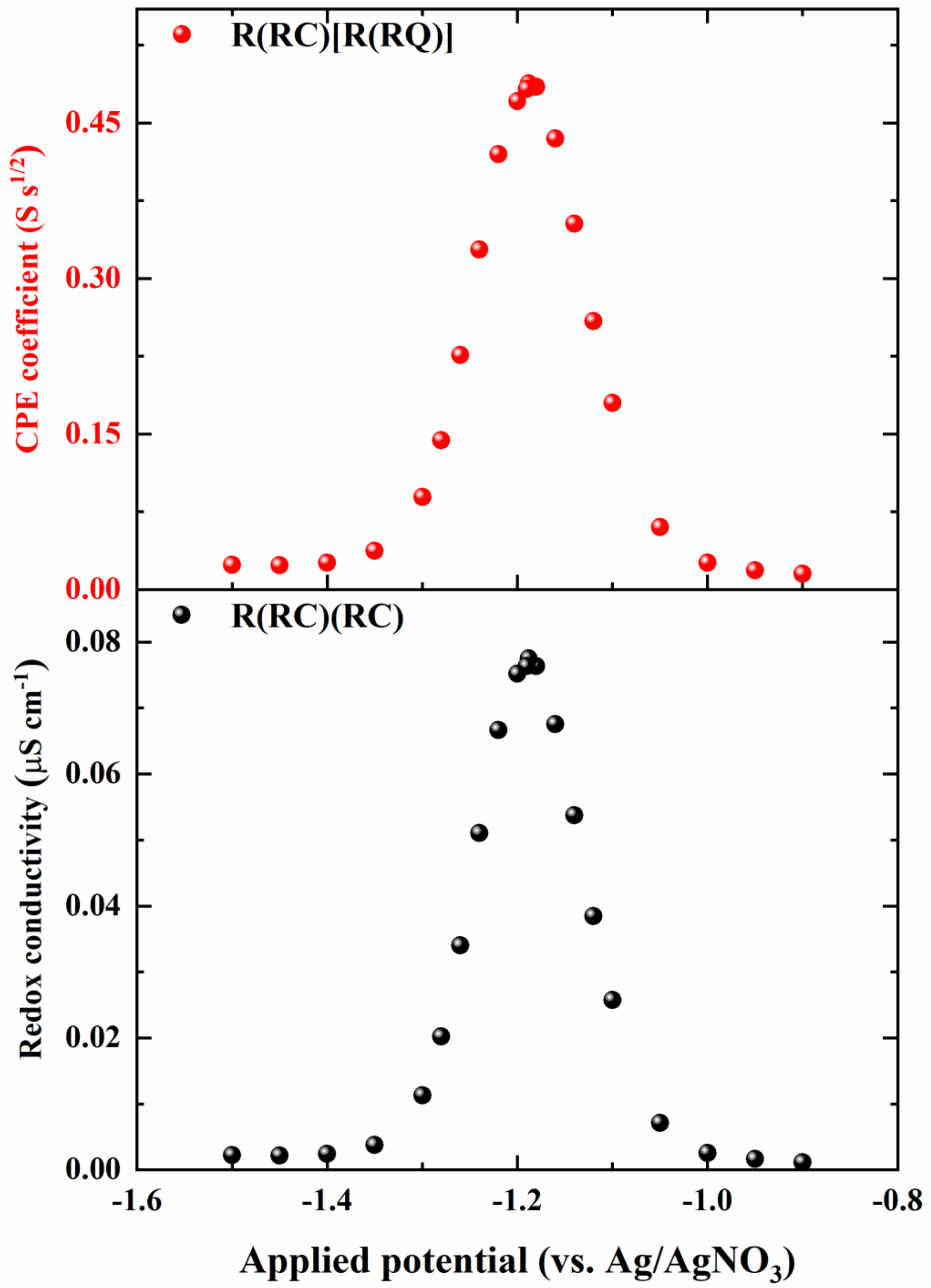

Supplementary Figure 32. Evolution of redox conductivity, as the function of electrochemical potential (redox state of the thin-film) measured in 0.1 M KPF_6 DMF electrolyte, which is determined by the mole fraction of electron reduction, *x*-UU-100(Co), $0.0 \leq x \leq 1.0$. Lower panel is simulated based on the circuit in **Supplementary Figure 31e**, and the upper panel is simulated based on the circuit in **Supplementary Figure 31f**. Note that the unit of the Y-axes for upper panel is changing upon the phase, *n*, to facilitate comparison, *n* was assumed to be 0.5 here.

10. Experimental EIS data of Zn(pyrazol-NDI) MOF thin-film at different redox states (Li^+ as counterions) and fittings with equivalent circuits

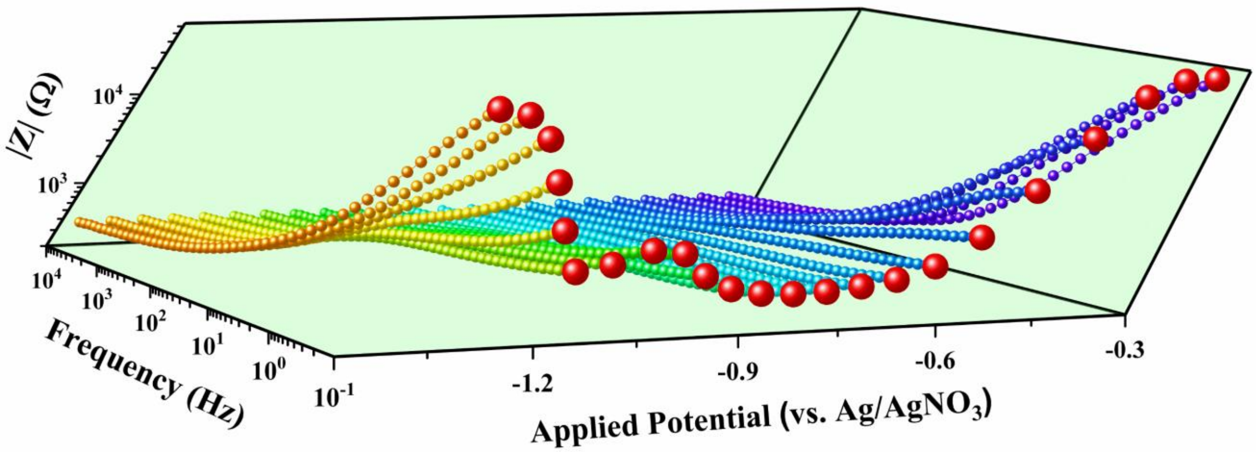

Supplementary Figure 33. Bode plots of Zn(pyrazol-NDI) thin-film at different applied potentials measured in 0.1 M $LiClO_4$ DMF electrolyte (the impedance data point at the frequency of 0.1 Hz was magnified for each measurement to highlight the effect of redox state), before each measurement, a stabilization time of 120 s was applied at respective potentials to achieve steady redox states.

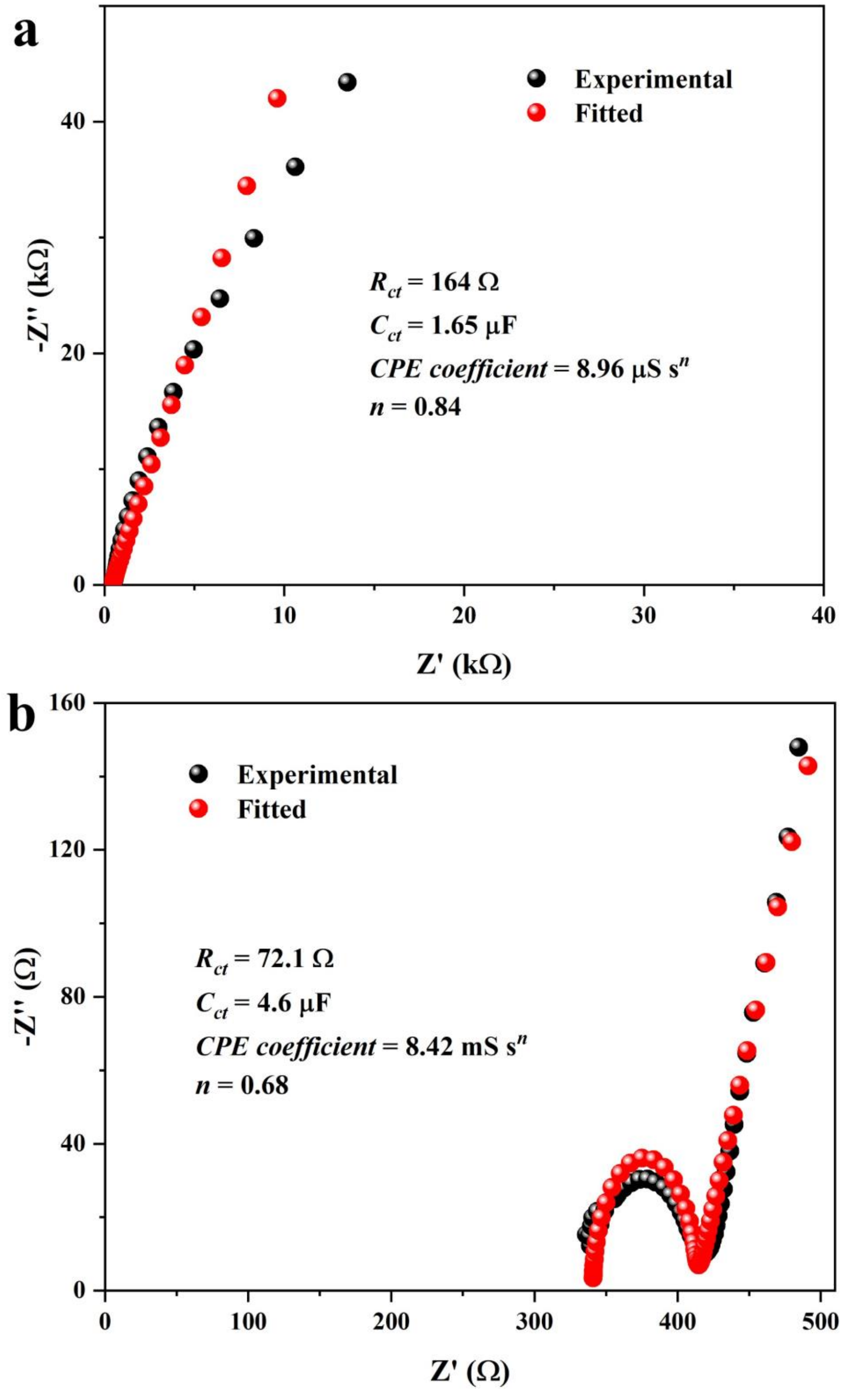

Supplementary Figure 34. Nyquist plots of experimental and fitted (circuit in **Supplementary Figure 11b**) electrochemical impedance data for *0.0*-Zn(pyrazol-NDI) (a) and *0.5*-Zn(pyrazol-NDI) (b) thin-films measured in 0.1 M $LiClO_4$ DMF electrolyte.

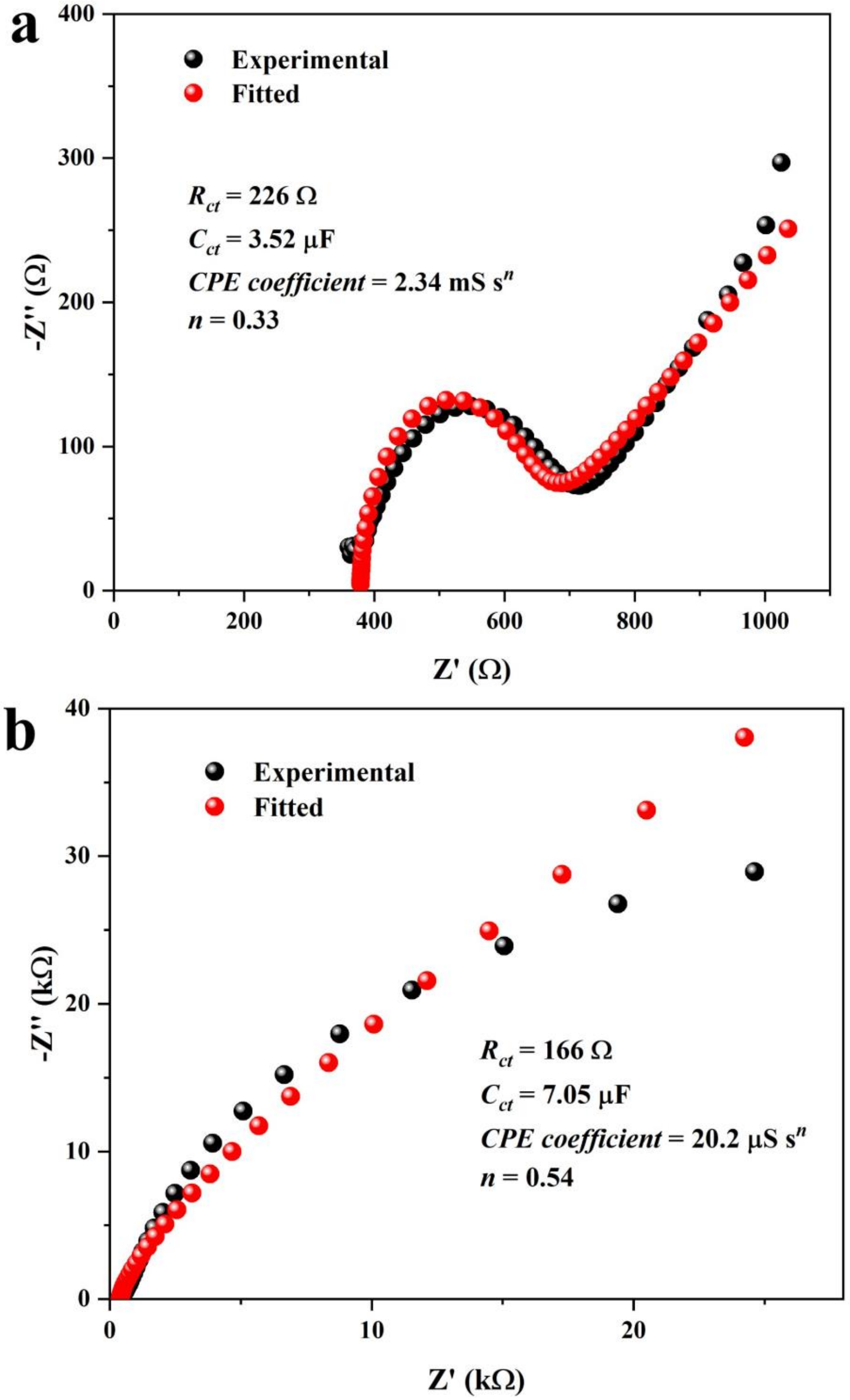

Supplementary Figure 35. Nyquist plots of experimental and fitted (circuit in **Supplementary Figure 11b**) electrochemical impedance data for *1.5*-Zn(pyrazol-NDI) (a) and *2.0*-Zn(pyrazol-NDI) (b) thin-films measured in 0.1 M $LiClO_4$ DMF electrolyte.

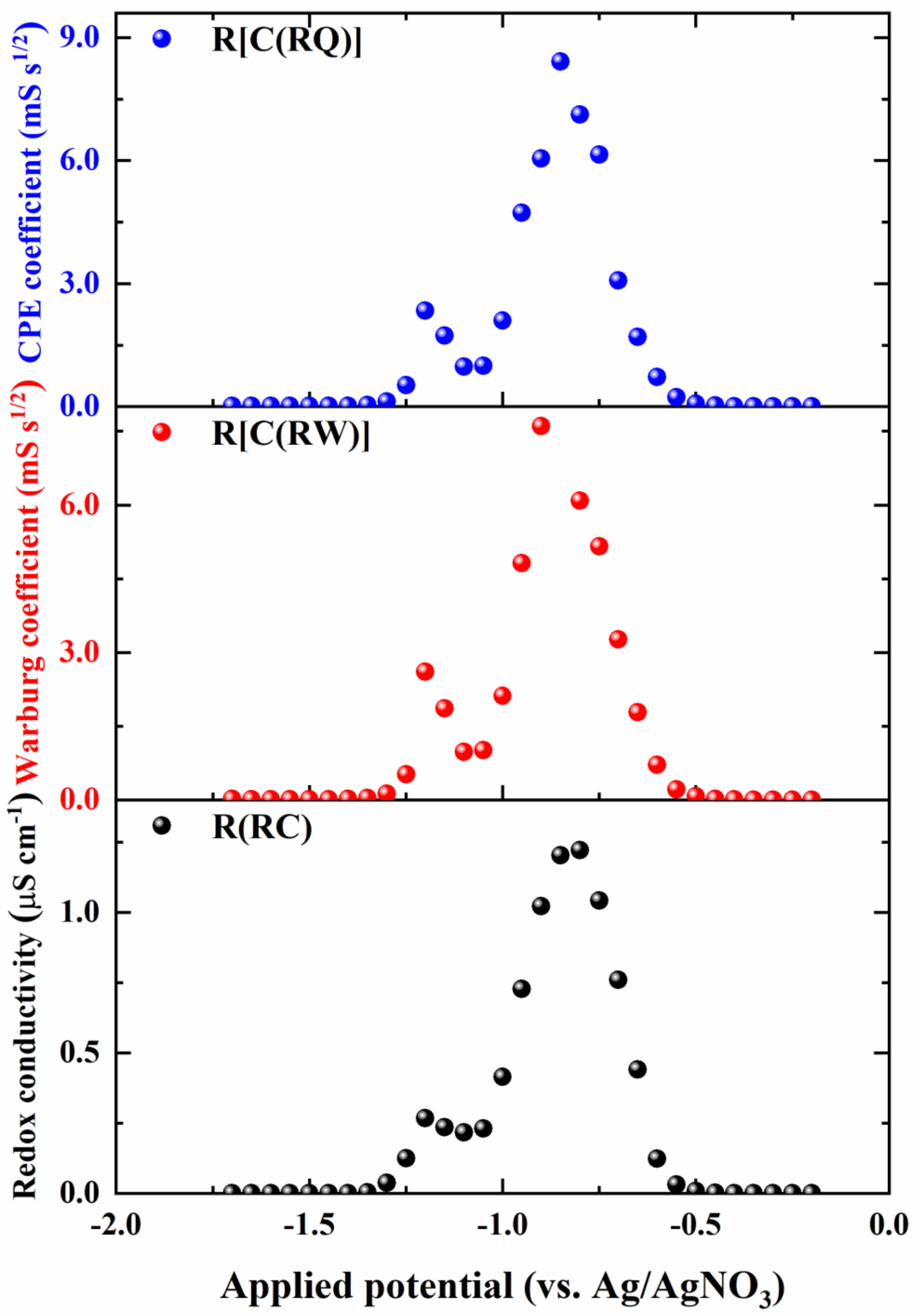

Supplementary Figure 36. Evolution of redox conductivity (lower panel, simulated from **Supplementary Figure 11a**), Warburg coefficient (middle panel, simulated from **Supplementary Figure 11c**) and CPE coefficient (upper panel, simulated from **Supplementary Figure 11b**) as the function of electrochemical potential (redox state of the thin-film) measured in 0.1 M $LiClO_4$ DMF electrolyte, which is determined by the mole fraction of electron reduction, x-Zn(pyrazol-NDI), $0.0 \leq x \leq 2.0$. Note that the unit of the Y-axes for upper panel is changing upon the phase, n, to facilitate comparison, n was assumed to be 0.5 here.

11. **Experimental EIS data of Zn(pyrazol-NDI) MOF thin-film at different redox states (TBA^+ as counterions) and fittings with equivalent circuits**

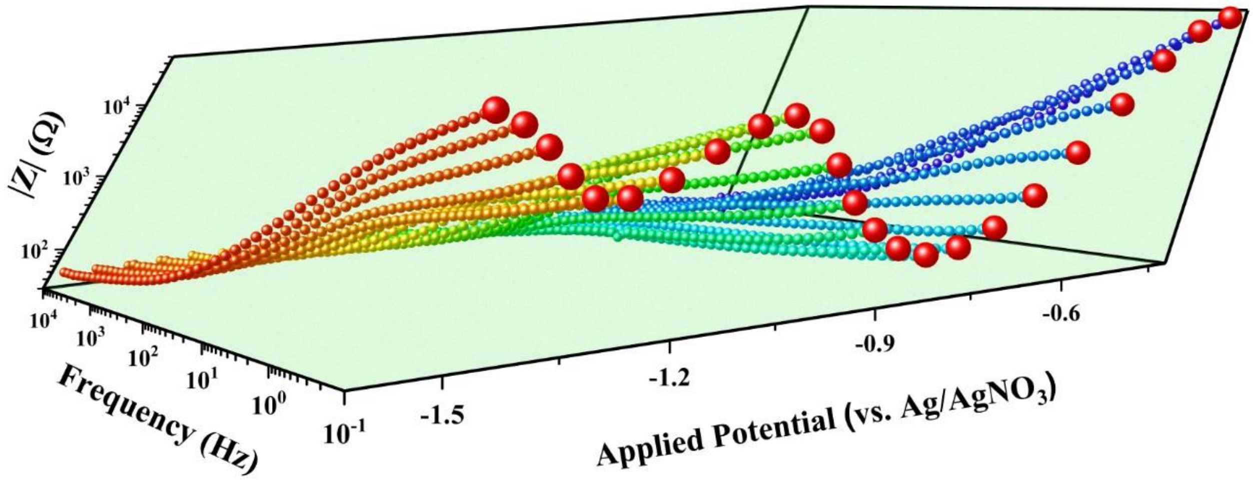

Supplementary Figure 37. Bode plots of Zn(pyrazol-NDI) thin-film at different applied potentials measured in 0.1 M $TBAPF_6$ DMF electrolyte (the impedance data point at the frequency of 0.1 Hz was magnified for each measurement to highlight the effect of redox state), before each measurement, a stabilization time of 120 s was applied at respective potentials to achieve steady redox states.

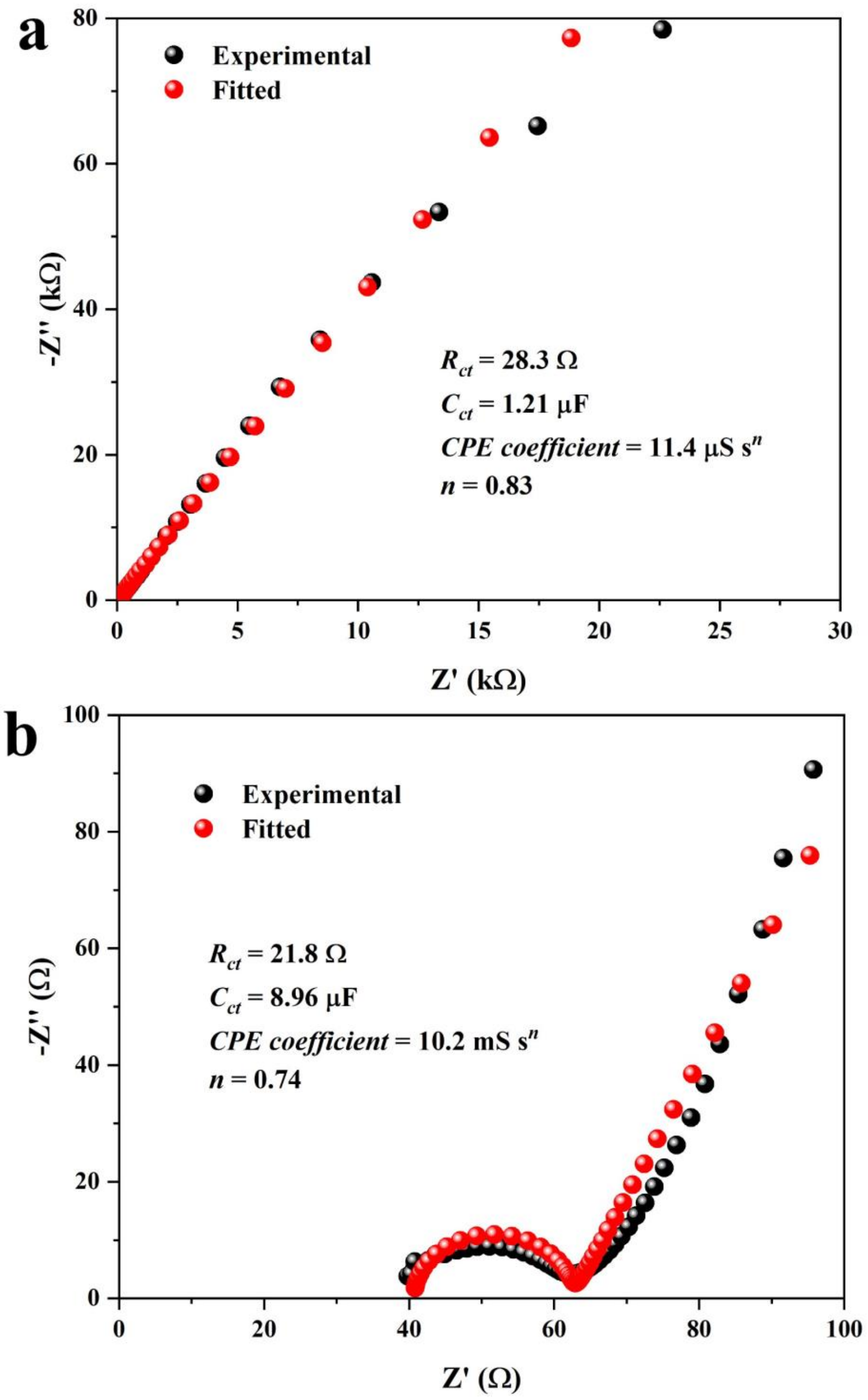

Supplementary Figure 38. Nyquist plots of experimental and fitted (circuit in **Supplementary Figure 11b**) electrochemical impedance data for *0.0*-Zn(pyrazol-NDI) (a) and *0.5*-Zn(pyrazol-NDI) (b) thin-films measured in 0.1 M $TBAPF_6$ DMF electrolyte.

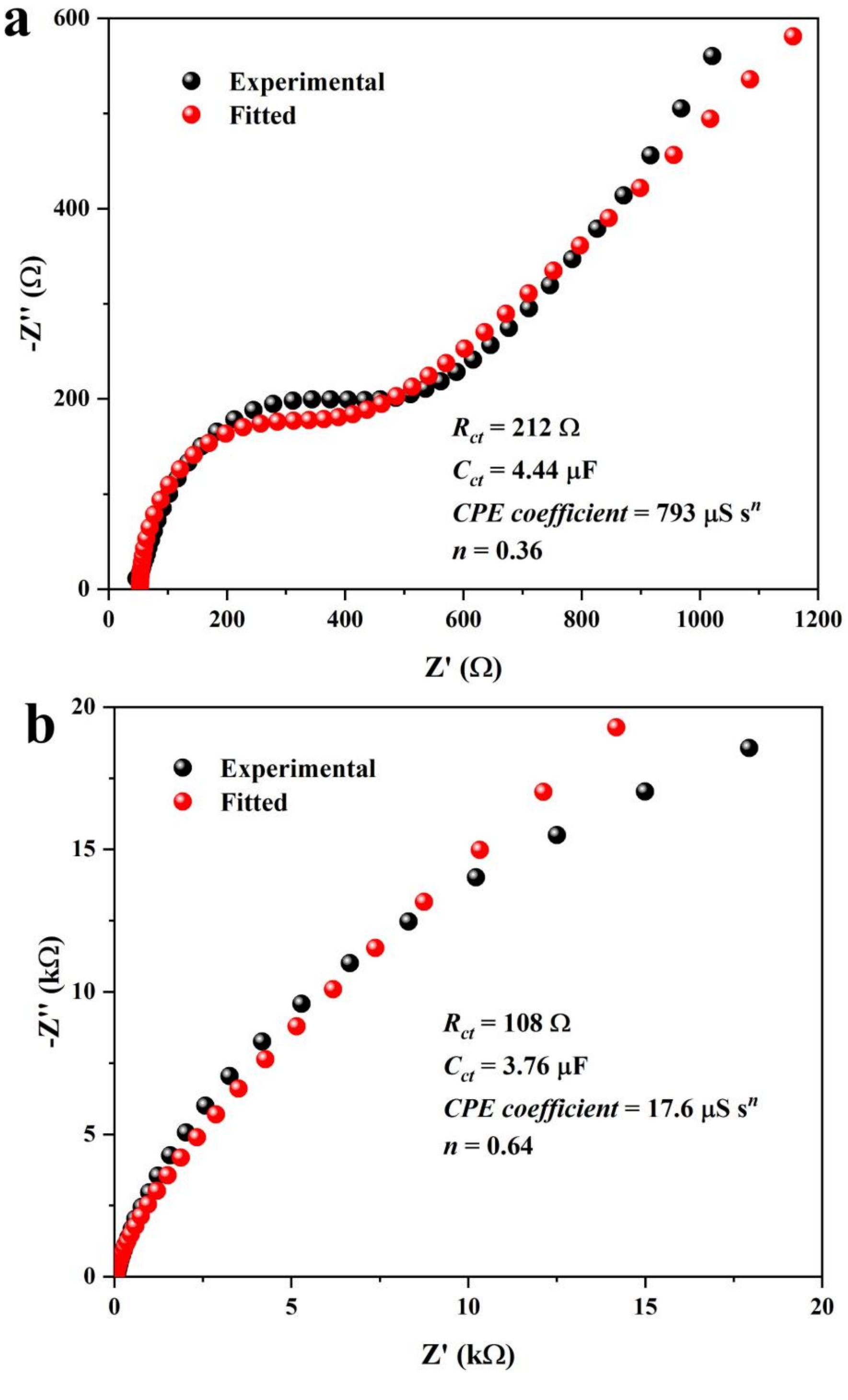

Supplementary Figure 39. Nyquist plots of experimental and fitted (circuit in **Supplementary Figure 11b**) electrochemical impedance data for *1.5*-Zn(pyrazol-NDI) (a) and *2.0*-Zn(pyrazol-NDI) (b) thin-films measured in 0.1 M $TBAPF_6$ DMF electrolyte.

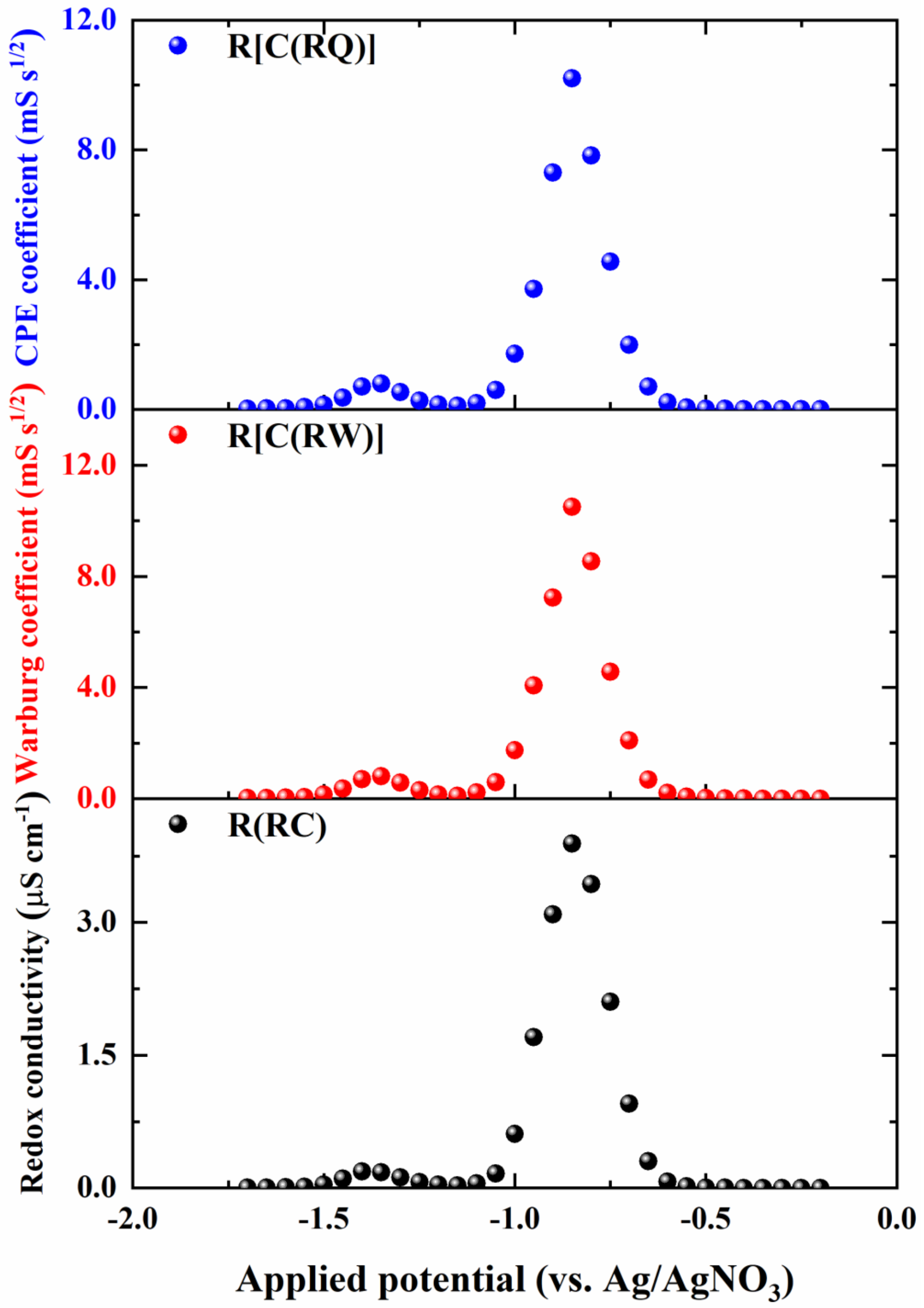

Supplementary Figure 40. Evolution of redox conductivity (lower panel, simulated from **Supplementary Figure 11a**), Warburg coefficient (middle panel, simulated from **Supplementary Figure 11c**) and CPE coefficient (upper panel, simulated from **Supplementary Figure 11b**) as the function of electrochemical potential (redox state of the thin-film) measured in 0.1 M $TBAPF_6$ DMF electrolyte, which is determined by the mole fraction of electron reduction, *x*-Zn(pyrazol-NDI), $0.0 \leq x \leq 2.0$. Note that the unit of the Y-axes for upper panel is changing upon the phase, *n*, to facilitate comparison, *n* was assumed to be 0.5 here.

12. Comparison of maximum redox conductivities and corresponding activation barriers of Zn(pyrazol-NDI) MOF thin-films as the function of counterions

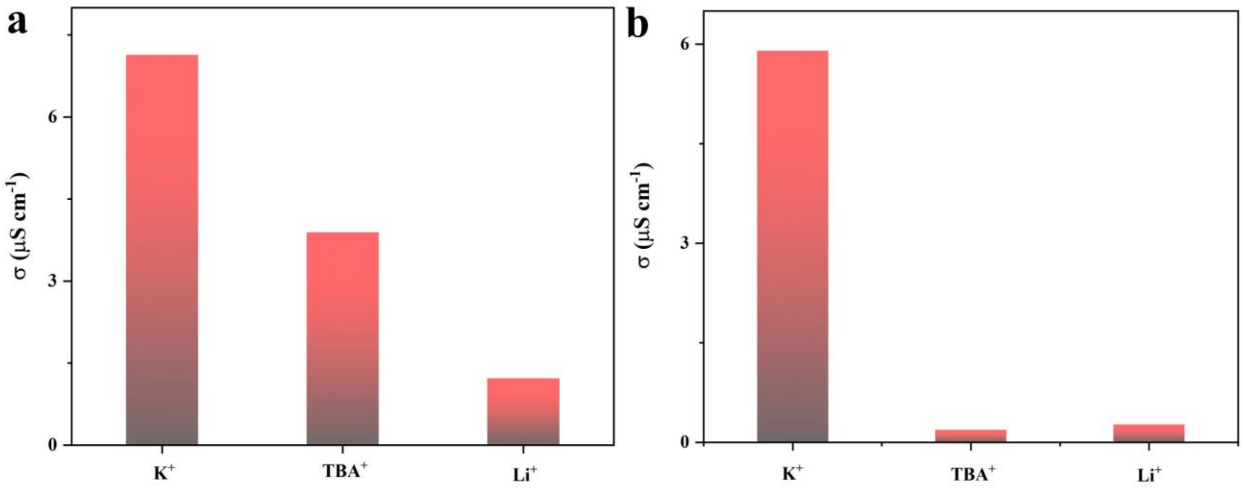

Supplementary Figure 41. Comparison of the maximum redox conductivities during the 1st (a) and 2nd (b) electron reduction of Zn(pyrazol-NDI) thin-films when using different counterions (K^+, Li^+ and TBA^+) as the supporting electrolyte (0.1 M).

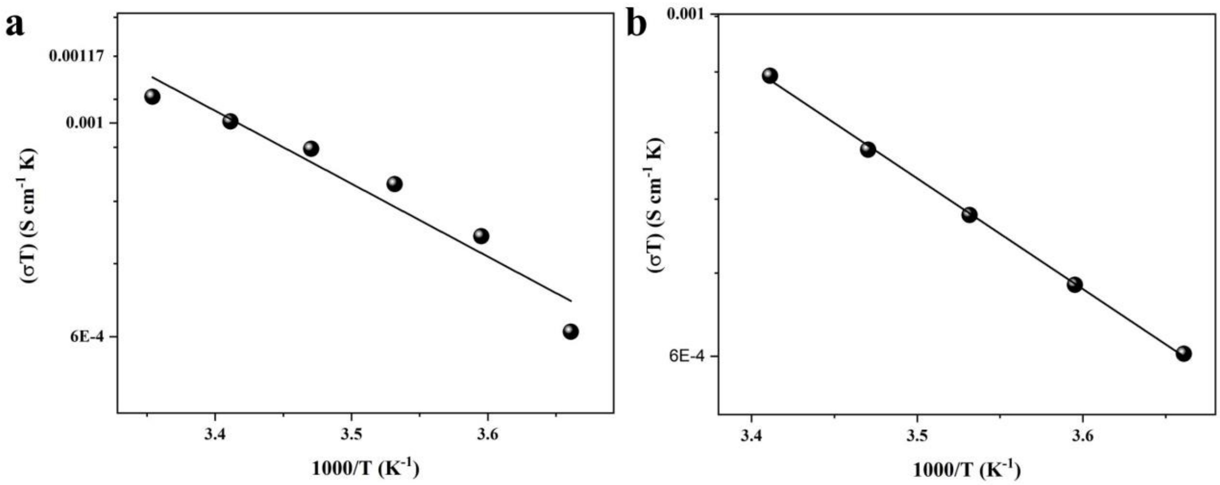

Supplementary Figure 42. Steady-state conductivity of *0.5*-Zn(pyrazol-NDI) thin-films as the function of temperature in the presence of Li^+ (a) and TBA^+ (b) as counterions. Activation energy is derived based on the Arrhenius equation.

13. Supplementary References

1 Costentin, C. & Nocera, D. G. Dual-Phase Molecular-like Charge Transport in Nanoporous Transition Metal Oxides. *J. Phys. Chem. C* **123**, 1966-1973, doi:10.1021/acs.jpcc.8b10948 (2018).

2 Saveánt, J. M. Electron Hopping Between Fixed Sites: Equivalent Diffusion and Migration Laws. *J. Electroanal. Chem. Interfacial Electrochem.* **201**, 211-213 (1986).

3 Wade, C. R., Corrales-Sanchez, T., Narayan, T. C. & Dincă, M. Postsynthetic tuning of hydrophilicity in pyrazolate MOFs to modulate water adsorption properties. *Energy Environ. Sci.* **6**, doi:10.1039/c3ee40876k (2013).
